# Global patterns of seasonal influenza activity, duration of activity and virus (sub)type circulation from 2010 to 2020

**DOI:** 10.1111/irv.12969

**Published:** 2022-02-24

**Authors:** Patrizio Zanobini, Guglielmo Bonaccorsi, Chiara Lorini, Mendel Haag, Ian McGovern, John Paget, Saverio Caini

**Affiliations:** ^1^ Department of Health Sciences University of Florence Florence Italy; ^2^ Center for Outcomes Research and Epidemiology Seqirus NL BV Amsterdam The Netherlands; ^3^ Center for Outcomes Research and Epidemiology Seqirus Inc Cambridge Massachusetts USA; ^4^ Netherlands Institute for Health Services Research (NIVEL) Utrecht The Netherlands

**Keywords:** duration of epidemics, influenza, influenza virus circulation, influenza virus type, subtype, and lineage, seasonal influenza, timing of epidemic peak

## Abstract

**Background:**

Seasonal influenza viruses undergo unpredictable changes, which may lead to antigenic mismatch between circulating and vaccine strains and to a reduced vaccine effectiveness. A continuously updated knowledge of influenza strain circulation and seasonality is essential to optimize the effectiveness of influenza vaccination campaigns. We described the global epidemiology of influenza between the 2009 A(H1N1)p and the 2020 COVID‐19 pandemic.

**Methods:**

Influenza virological surveillance data were obtained from the WHO‐FluNet database. We determined the median proportion of influenza cases caused by the different influenza virus types, subtypes, and lineages; the typical timing of the epidemic peak; and the median duration of influenza epidemics (applying the annual average percentage method with a 75% threshold).

**Results:**

We included over 4.6 million influenza cases from 149 countries. The median proportion of influenza cases caused by type A viruses was 75.5%, highest in the Southern hemisphere (81.6%) and lowest in the intertropical belt (73.0%), and ranged across seasons between 60.9% in 2017 and 88.7% in 2018. Epidemic peaks typically occurred during winter months in Northern and Southern hemisphere countries, while much more variability emerged in tropical countries. Influenza epidemics lasted a median of 25 weeks (range 8–42) in countries lying between 30°N and 26°S, and a median of 9 weeks (range 5–25) in countries outside this latitude range.

**Conclusions:**

This work will establish an important baseline to better understand factors that influence seasonal influenza dynamics and how COVID‐19 may have affected seasonal activity and influenza virus types, subtypes, and lineages circulation patterns.

## 1 INTRODUCTION

Influenza is a major respiratory disease that causes significant morbidity and mortality globally.^1^, ^2^ Influenza vaccination is the primary intervention to prevent influenza infections and their complications, thus deploying the vaccine efficiently and in a timely manner with regard to the timing of occurrence of influenza epidemics is critical in order to limit the burden of disease of influenza.

The World Health Organization (WHO) has established the Global Influenza Surveillance and Response System (GISRS) with the aim to conduct epidemiological and virological surveillance of influenza globally.^3^ The GISRS is instrumental for the WHO influenza vaccine committee in order to advise on the antigenic composition of influenza vaccines for the next influenza season.^4^, ^5^ For the Northern hemisphere, influenza virus circulation patterns are reviewed every February, so that the vaccine can be distributed in early autumn, in advance of the winter influenza season. Recommendations for the Southern Hemisphere are usually issued in September, so that the vaccine can be deployed in advance of the immunization campaigns of March–April of the following year. The overall goal is to optimize vaccine formulation and timing of administration to obtain maximal vaccine effectiveness in all countries worldwide. This is particularly challenging, however, for tropical regions, where influenza viruses often circulate year‐round and the periods of influenza activity can vary even between neighbouring countries or countries at the same latitude.^6^, ^7^, ^8^ The timing of the vaccination is especially critical when considering that vaccine‐induced immunity may wane during an influenza season, particularly in the elderly.^9^ The picture is further complicated by the frequent, yet largely unpredictable changes in circulating influenza virus strains, which may lead to antigenic mismatch between circulating strains and vaccine strains and ultimately to a reduced vaccine effectiveness.^10^, ^11^, ^12^ Finally, the regular seasonal dynamics can be greatly altered by the emergence of new pandemic influenza viruses, as was the case in 2009. More recently, Sars‐Cov‐2 virus circulation has caused a dramatic reduction in influenza circulation worldwide, most likely as a result of non‐pharmaceutical interventions and reduction of global travel.^13^, ^14^

The aim of our study was to perform an updated analysis of the global epidemiology of seasonal influenza for the period 2010–2020, describing the patterns of circulation of the different influenza virus types, subtypes, and lineages, and determining the typical peak timing and duration of influenza epidemics for countries in temperate (Northern/Southern hemispheres) and tropical climate regions. While influenza activity is currently low, influenza will become a public health issue again but the timing and severity of future influenza outbreaks is hard to foresee at the moment. Having a full picture of the epidemiology of influenza in the decade preceding the COVID‐19 pandemic (defined as the period from the 2009–2010 H1N1pdm09 influenza pandemic and the emergence of the COVID‐19 pandemic in early 2020) will establish an important baseline to better understand factors that influence annual influenza dynamics.

## 2 MATERIAL AND METHODS

### 2.1 Data source and definitions

Influenza virological surveillance data were obtained from the publicly available web‐based database FluNet, which is coordinated by the WHO.^15^ Information on the weekly number of laboratory‐confirmed cases of influenza (overall and by virus type, type A subtype, and type B lineage) is entered into the FluNet database by the national influenza centres and other influenza reference laboratories of 194 states participating in the Global Influenza Surveillance and Response System (GISRS) or are uploaded from WHO regional databases. On 22 April 2021, we downloaded the weekly number of laboratory‐confirmed influenza cases reported to the national surveillance systems of all WHO Regions between week 1/2010 and week 52/2020, broken down by virus type (influenza A, B), type A subtype (H1N1, H3N2, other subtypes, unsubtyped), and type B lineage (Victoria, Yamagata, uncharacterized). Because influenza A virus subtypes other than H1N1 and H3N2 were very rarely reported (H5N1 and pre‐pandemic H1N1 subtype accounted for around 1 out of 10,000 of total cases in the study database), these were merged into a single category with unsubtyped type A influenza cases.

All statistical analyses were conducted by country, and by grouping countries according to either of two geographical criteria: countries belonging to each of the six WHO regions (Africa, Eastern Mediterranean, European, South‐East Asia, Western Pacific, and Americas); or countries situated in the Northern hemisphere (centroid lying north of the tropic of Cancer, latitude 23°27′ N), in the Southern hemisphere (centroid lying south of the tropic of Capricorn, latitude 23°27′ S), or in the intertropical belt (centroid lying between the two tropics) (this categorization will be referred to as “latitudinal area” henceforth). For the latter classification, the geographical coordinates (latitude and longitude) of country centroids (i.e., central points) were downloaded from the Harvard WorldMap webpage in the month of May.^16^

The unit of analysis was the “season,” which was defined as the calendar year (from week 1 to week 52/53) in countries situated in the Southern hemisphere and the intertropical belt, or from week 27 of a year to week 26 of the next year for northern hemisphere countries (where influenza epidemics, which typically occur in autumn and winter months, can often bridge two consecutive calendar years). In what follows, the expression “season 2013” will therefore correspond to the calendar year 2013 for countries in the Southern hemisphere and in the intertropical belt, and to the period from week 27/2013 to week 26/2014 for countries in the Northern hemisphere. Considering that data were downloaded from week 1/2010 to week 52/2020 for all countries, the maximum number of seasons that could be included in the analyses was 11 (from 2010 to 2020) for Southern hemisphere and intertropical belt countries, and 10 (from season 2010–2011 to season 2019–2020) for Northern hemisphere countries (i.e., data from weeks 1–26 of 2010 and weeks 27–52 of 2020 were not used for Northern hemisphere countries). Countries with fewer than 50 influenza cases in a given season were excluded from the analysis of that season in order to enhance robustness to the study results.^17^

### 2.2 Circulation patterns of influenza viruses

For each country and season, we determined the proportion of influenza cases that were caused by either virus type (A and B). We then calculated its median value, and the proportion of seasons in which either influenza virus type accounted for ≥80%, ≥50 to 80%, ≥20 to 50%, or < 20% of all reported influenza cases, in each country and for countries belonging to each latitudinal area and WHO region. Furthermore, for each season in each country we determined the proportion of influenza cases that were caused by each type A virus subtype (H1N1, H3N2, and other/unsubtyped) and type B virus lineage (Victoria, Yamagata, and uncharacterized). The non‐parametric Kruskal–Wallis test was applied to compare median proportions between countries belonging to different latitudinal areas or WHO regions.

### 2.3 Peak timing and duration of influenza epidemics

The determination of the timing of the primary and secondary peak of influenza circulation was conducted by analysing country‐specific influenza times‐series using the EPIPOI software.^18^ Because our objective was to define the “typical” timing of the epidemic peak, we included only countries with five or more seasons with at least 50 reported influenza cases. The season 2020 (i.e., the calendar year for countries in the Southern hemisphere and the intertropical belt, and the season 2020–2021 for Northern hemisphere countries) was not included in this analysis because it was particularly atypical due to the COVID‐19 pandemic. In addition, because EPIPOI needs the same number of time points (in our case weeks) to operate, we excluded all 53rd weeks in our dataset (which only applied to years 2014 and 2020). EPIPOI proceeds by first detrending the country‐specific time series using a quadratic polynomial, and it then works out the periodic annual function (PAF) of the time series by summing up the annual, semi‐annual, and quarterly harmonics as obtained by Fourier decomposition. The typical timing of the peak corresponds to the peak month of the PAF. The amplitude of the PAF is obtained as the ratio of the wave height to the peak value and can be interpreted as an estimate of the intensity of seasonality of influenza epidemics in a given country (i.e., as a measure of how much influenza cases in a given country tend to occur over a short period of time instead of being distributed over the entire season). Owing to how it is calculated, the amplitude can sometimes take values above 100%, particularly when the time‐series fall to zero for a certain number of weeks, for example, in summer months of temperate countries. (for more details, refer to Alonso and McCormick, 2012^19^).

The duration of the influenza epidemic in each season was defined using the average annual percentage (AAP) method, according to which the duration is defined as the shortest stretch of consecutive weeks that account for at least 75% of all influenza cases that were reported in the season.^20^ As for the determination of the typical timing of the epidemic peak, this analysis was conducted by including only the 122 countries that have data available for five or more seasons with ≥50 reported cases, and after discarding the season 2020 because of the COVID‐19 pandemics.

### 2.4 Software

All analyses were conducted using Stata version 15 (Stata Corp, College Station, TX) and the freely available analytical software EPIPOI.^18^, ^19^

## 3 RESULTS

### 3.1 Circulation patterns of influenza viruses

In total, data were available for 149 countries (77 in the Northern hemisphere, 66 in the intertropical belt, and six in the Southern hemisphere) which contributed a total of 1244 seasons encompassing 4,659,001 influenza cases. The median number of seasons included per country was 10, and the median number of reported influenza cases per season was 494.

Type A and B influenza viruses accounted for 72.5% and, respectively, 27.5% of all influenza cases reported globally between 2010 and 2020 (Table S1). The median percentage of influenza cases that were caused by type A viruses was 75.5%. More in detail, the proportion of all reported influenza cases that were caused by type A viruses was ≥80% in 42.3% of seasons, ≥50% and <80% in 42.3% of seasons, ≥20% and <50% in 13.7% of seasons, and <20% in only 1.7% of seasons. These figures varied somewhat geographically (*p* value for difference of the median proportion of A cases between latitudinal areas <0.001) (Table 1). The median proportion of influenza A cases and the percentage of seasons in which type A influenza viruses caused ≥80% cases were lowest in countries of the intertropical belt (73% and 38.8%, respectively, based on data from 66 countries and 528 seasons), and highest in countries in the Southern hemisphere (81.6% and 55.6%, based on data from six countries and 63 seasons). When grouping countries according to the six WHO regions (Table 2), type A influenza viruses accounted for the largest share of all influenza cases in the Region of the Americas and the European region (median % of type A cases was 80.8% and 77.2%, respectively), and for the smallest share in the Africa and the South‐East Asia regions (where a median of 69.1% and 68.1% of all influenza cases were caused by type A viruses); the *p* value for difference of medians between WHO regions was <0.001. Differences in the circulation of type A and B influenza viruses were observed also by season (Table 3). Influenza type A viruses accounted for a minimum of 60.9% of all influenza cases (median value calculated across all countries) in the season 2017, and a maximum of 88.7% of cases in the season 2018. Further variability was observed when stratifying by season and geographical area (Table S2).

**TABLE 1:** Circulation of type A and B influenza viruses in countries lying in the northern hemisphere, intertropical belt, or southern hemisphere

Geographical area	*N* seasons (≥50 cases)	*N* influenza cases to reported to FluNet^a^	Influenza type A		Influenza type B		Median reported cases per season	Median % A	Country seasons with % A			
			*N*	%	*N*	%			≥80%	≥50% to <80%	≥20% to <50%	<20%
Northern hemisphere	653	4,212,382	3,042,293	72.2%	1,170,089	27.8%	835	76.3%	287	260	93	13
Intertropical belt	528	285,964	212,589	74.3%	73,375	25.7%	271	73.0%	205	241	74	8
Southern hemisphere	63	160,655	124,317	77.4%	36,338	22.6%	1882	81.6%	35	25	3	0
Total	1.244	4,659,001	3,379,199	72.5%	1,279,802	27.5%	494	75.0%	527 (42.3%)	526 (42.3%)	170 (13.7%)	21 (1.7%)

*Source*: WHO FluNet database 2010–2020. Only seasons with ≥50 reported influenza cases overall were included in the analysis. See text for details.

^a^ Surveillance cases do not reflect the incidence rate of influenza.

**TABLE 2:** Circulation of type A and B influenza viruses by WHO region

WHO region	*N* season (≥50 cases)	*N* influenza cases reported to FluNet^a^	Influenza type A		Influenza type B		Median cases reported per season	Median % A	Country seasons with % A			
			*N*	%	*N*	%			≥80%	≥50% to <80%	≥20% to <50%	<20%
African region	202	62,735	42,336	67.5%	20,399	32.5%	211	69.1%	61	105	34	2
Eastern Mediterranean region	118	129,350	99,074	76.6%	30,276	23.4%	407	76.8%	50	59	9	0
European region	437	1,409,435	1,023,248	72.6%	386,187	27.4%	879	77.2%	203	151	71	12
Region of the Americas	262	2,068,582	1,520,028	73.5%	548,554	26.5%	384	80.8%	136	100	24	2
South‐East Asia region	85	90,632	67,838	74.8%	22,794	25.2%	722	68.1%	26	46	11	2
Western Pacific region	140	898,267	626,675	69.8%	271,592	30.2%	768	70.3%	51	65	21	3
Total	1244	4,659,001	3,379,199	72.5%	1,279,802	27.5%	494	75.0%	527 (42.3%)	526 (42.3%)	170 (13.7%)	21 (1.7%)

*Source*: WHO FluNet database 2010–2020. Only seasons with ≥50 reported influenza cases overall were included in the analysis. See text for details.

^a^ Surveillance cases do not reflect the incidence rate of influenza.

**TABLE 3:** Circulation of type A and B influenza viruses by season from 2010 to 2020

Season^a^	*N* season (≥50 cases)	*N* influenza cases reported to FluNet^b^	Influenza type A		Influenza type B		Median cases per season	Median % A	Country seasons with % A			
			*N*	%	*N*	%			≥80%	≥50% to <80%	≥20% to <50%	<20%
2010	109	283,943	214,546	75.6%	69,397	24.4%	703	78.7%	51	43	11	4
2011	104	183,551	129,362	70.5%	54,189	29.5%	497	81.9%	59	28	16	1
2012	115	307,187	214,768	69.9%	92,419	30.1%	527	64.9%	27	66	22	0
2013	110	283,798	224,395	79.1%	59,403	20.9%	467	84.8%	60	41	9	0
2014	114	425,242	323,599	76.1%	101,643	23.9%	534	71.3%	32	64	17	1
2015	116	445,091	297,107	66.7%	147,984	33.3%	504	74.9%	49	48	17	2
2016	129	504,020	406,294	80.6%	97,726	19.4%	434	83.3%	73	39	17	0
2017	131	836,232	492,167	58.9%	344,065	41.1%	572	60.9%	24	58	39	10
2018	133	687,766	626,414	91.1%	61,352	8.9%	564	88.7%	82	45	5	1
2019	140	693,016	443,713	64.0%	249,303	36.0%	489	73.2%	50	77	12	1
2020	43	9155	6834	74.6%	2321	25.4%	134	75.0%	20	17	5	1
Total	1244	4,659,001	3,379,199	72.5%	1,279,802	27.5%	494	75.0%	527 (42.3%)	526 (42.3%)	170 (13.7%)	21 (1.7%)

*Source*: WHO FluNet database 2010–2020. Only seasons with ≥50 reported influenza cases overall were included in the analysis. See text for details.

^a^ Season is defined as the calendar year (from week 1 to week 52/53) in countries situated in the southern hemisphere and the intertropical belt, or from week 27 of a year to week 26 of the next year for northern hemisphere countries.

^b^ Surveillance cases do not reflect the Incidence of Influenza.

The proportion of influenza virus type A subtypes (H1N1, H3N2 and other/unsubtyped) and type B lineages (Victoria, Yamagata, and uncharacterized) in relation to the total number of influenza cases in each season and country is reported in Table S3. The proportion of influenza A cases that were subtyped was 56.6%, while 23.4% of all influenza B cases were characterized. The median % of cases accounted for in a season was 29.5% for A(H3N2), 23.4% for A(H1N1), 13.8% for other/unsubtyped A, 5.8% for B Yamagata, 3.3% for B Victoria, and 22.8% for uncharacterized B globally. A(H3N2) and A(H1N1) were the most commonly reported subtype/lineage in five and six seasons globally, respectively (Table 4).

**TABLE 4:** Circulation of the different influenza virus types, subtypes, and lineages by season

Season	*N* country season (≥50 cases)	*N* influenza cases reported to FluNet^a^	A(H3N2)		A(H1N1)		A other or unsubtyped		B Yamagata		B Victoria		B uncharacterized	
			*N*	%	*N*	%	*N*	%	*N*	%	*N*	%	*N*	%
2010	109	28,3943	56,844	20.0%	114,658	40.4%	43,044	15.2%	1704	0.6%	5699	2.0%	61,994	21.8%
2011	104	18,3551	73,037	39.8%	24,647	13.4%	31,675	17.3%	4893	2.7%	10,969	6.0%	38,327	20.9%
2012	115	30,7187	93,830	30.5%	58,734	19.1%	61,916	20.2%	8800	2.9%	2591	0.8%	81,028	26.4%
2013	110	28,3798	60,947	21.5%	111,731	39.4%	51,670	18.2%	10,780	3.8%	2238	0.8%	46,385	16.3%
2014	114	42,5242	174,607	41.1%	21,440	5.0%	127,489	30.0%	22,499	5.3%	1056	0.2%	78,088	18.4%
2015	116	44,5091	50,476	11.3%	145,073	32.6%	101,319	22.8%	15,531	3.5%	32,302	7.3%	100,151	22.5%
2016	129	50,4020	172,186	34.2%	38,331	7.6%	195,332	38.8%	11,647	2.3%	11,436	2.3%	74,643	14.8%
2017	131	83,6232	138,493	16.6%	91,530	10.9%	261,962	31.3%	68,171	8.2%	9099	1.1%	266,795	31.9%
2018	133	68,7766	101,151	14.7%	181,758	26.4%	343,038	49.9%	5757	0.8%	25,774	3.7%	29,821	4.3%
2019	140	69,3016	87,856	12.7%	106,481	15.4%	249,126	35.9%	2331	0.3%	45,045	6.5%	201,927	29.1%
2020	43	9155	1551	16.9%	3886	42.4%	1394	15.2%	45	0.5%	909	9.9%	1367	14.9%
Total	1244	4,659,001	1,010,978	21.7%	898,269	19.3%	1,467,965	31.5%	152,158	3.3%	147,118	3.2%	980,526	21.0%

*Source*: WHO FluNet database 2010–2020. Only seasons with ≥50 reported influenza cases overall were included in the analysis. See text for details.

^a^ Surveillance cases do not reflect the incidence rate of influenza.

### 3.2 Peak timing and duration of influenza epidemics

**FIGURE 1:**
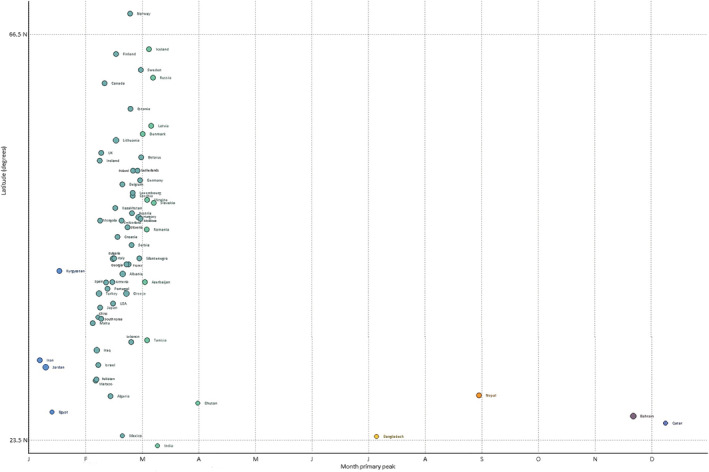
Typical timing of the primary peak of influenza detection in north hemisphere by country, against the latitudinal position of the country centroid. The size of the circles is proportional to the amplitude of influenza seasonality. *Source*: WHO FluNet database 2010–2019. Only countries with ≥5 seasons with ≥50 reported influenza cases were included in the analysis. See text for details

**FIGURE 2:**
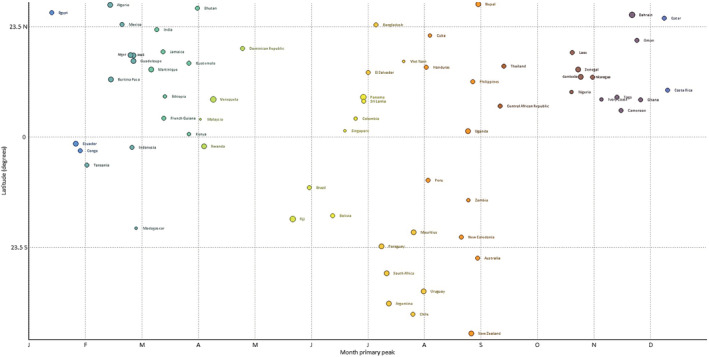
Typical timing of the primary peak of influenza detection in the intertropical belt and in the southern hemisphere by country, against the latitudinal position of the country centroid. The size of the circles is proportional to the amplitude of influenza seasonality. WHO FluNet database 2010–2019. Only countries with ≥5 seasons with ≥50 reported influenza cases were included in the analysis. See text for details

**FIGURE 3:**
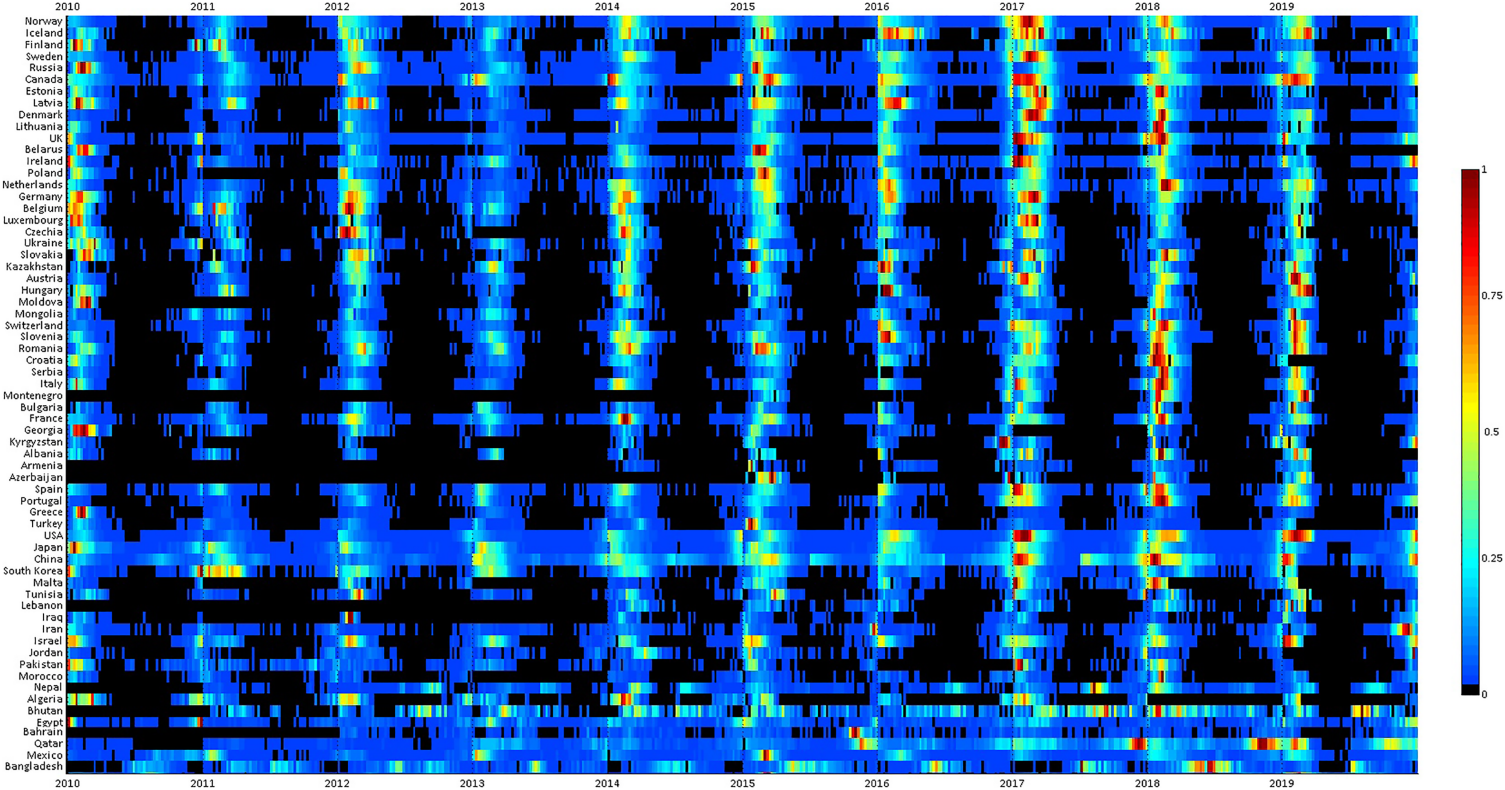
Heatmap of influenza virus detections in north hemisphere sorted by latitude of the country centroid. Colour bar represents the intensity of influenza activity, from high (red) to low (blue). Monthly incidence counts were standardized annually, and shown as the proportion of the maximum number of cases in a month for that country and period. WHO FluNet database 2010–2019. Only countries with ≥5 seasons with ≥50 reported influenza cases were included in the analysis. See text for details

**FIGURE 4:**
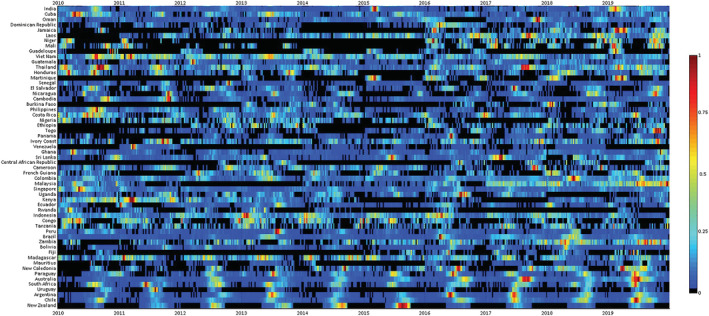
Heatmap of influenza virus detections in the intertropical belt and south hemisphere sorted by latitude of the country centroid. Colour bar represents the intensity of influenza incidence activity, from high (red) to low (blue). Monthly incidence counts were standardized annually, and shown as the proportion of the maximum number of cases in a month for that country and period. WHO FluNet database 2010–2019. Only countries with ≥5 seasons with ≥50 reported influenza cases were included in the analysis. See text for details

A total of 122 countries with at least five seasons with ≥50 reported influenza cases between 2010 to 2019 were included. The typical timing of the primary epidemic peak against the country latitude was depicted graphically in Figures 1 and 2 (the corresponding heat maps, showing the distribution of cases in each country over the study period, is provided as Figures 3 and 4). For Northern hemisphere countries, the typical timing of the epidemic peak fell between the beginning of December and the end of March (the vast majority occurring in February and the first half of March), while for Southern hemisphere countries, the epidemic peak typically took place in July or August. Much more variability emerged among countries in the intertropical belt, which encompassed countries whose typical epidemic peak could occur at any time of the year. Unlike countries in the Northern and Southern hemispheres, some of those situated in the intertropical belt were characterized by small‐amplitude primary peaks and well‐defined (amplitude above 50%) secondary peaks. The typical timing of the primary and secondary peak of influenza epidemics in each country included in the analyses are available in Table S4.

**FIGURE 5:**
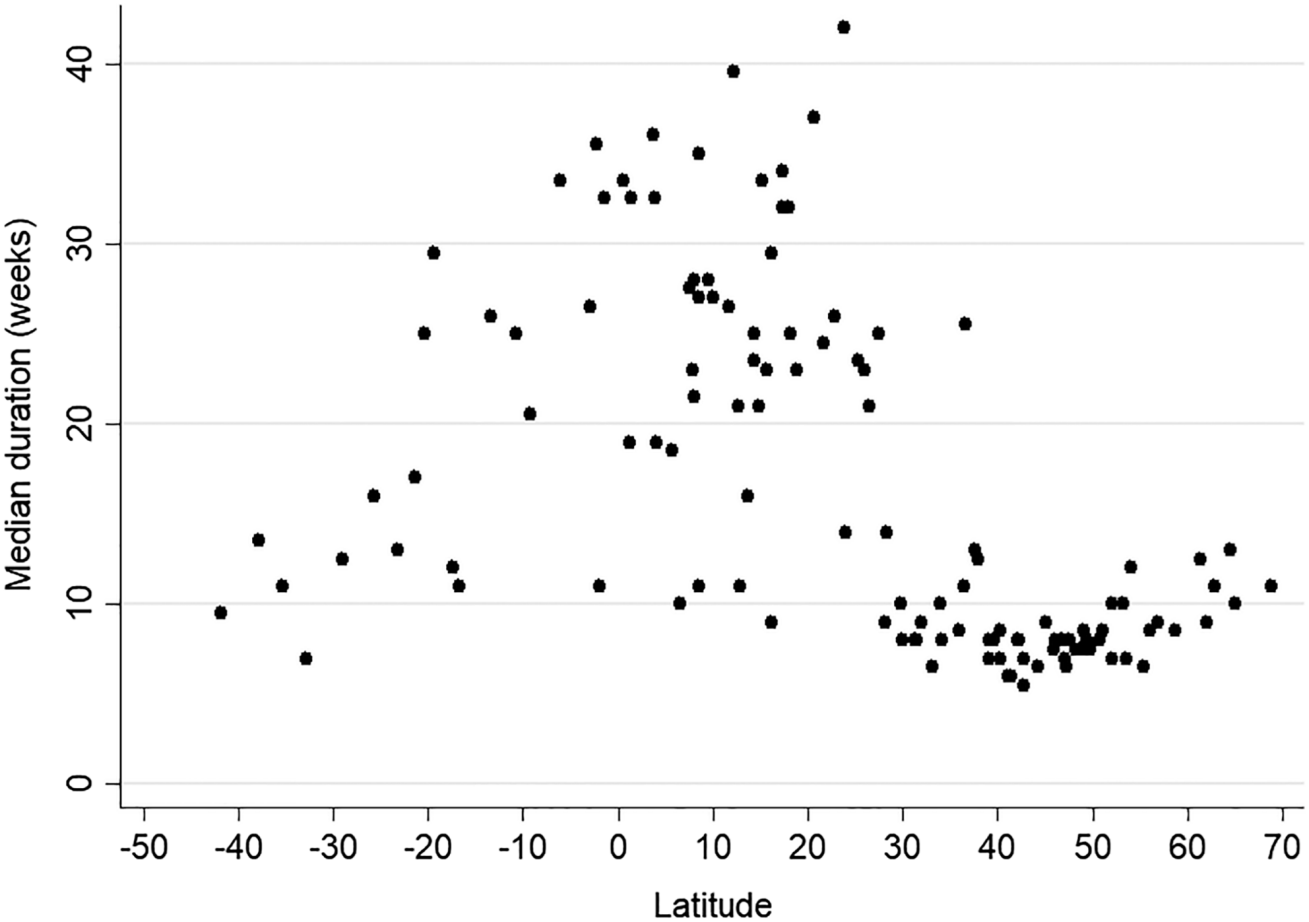
Median duration of the influenza epidemics (in weeks) by country latitude. The duration of the season was calculated according to the average annual percentage (AAP) method, with a 75% threshold. WHO FluNet database 2010–2019. Only countries with ≥5 seasons with ≥50 reported influenza cases were included in the analysis. See text for details

The association between the median duration of the influenza epidemics and the country's latitude is depicted in Figure 5, and the complete data are available in Table S4. By visually inspecting Figure 5, it was evident that influenza epidemics had a shorter duration at the two extremes of the latitude range. In particular, the median duration of the influenza epidemics was shorter than 15 weeks for the 60 countries lying at a latitude above 30°N and below of 26°S (the choice of these two cut‐offs was data driven and not made a priori); the only exception is China, a vast country that stretches across a large range of latitudes. The 62 countries whose centroid was situated between 30°N and 26°S showed a substantial variability in the duration of the influenza epidemics: the median duration of influenza epidemics in this range of latitudes was ≤15 weeks for 13 countries, between 16 and 29 weeks for 34 countries, and ≥30 weeks for 15 countries. Of note, there was a mild, yet statistically significant positive association between the median duration of influenza epidemics and the country latitude for countries whose centroid was situated at latitudes above 40°N (the more northern the country, the longer duration of influenza epidemics, *p* value <0.001).

## 4 DISCUSSION

We described the global variability in the circulation of influenza virus strains and in the timing and duration of influenza epidemics between two pandemic periods of the 21st century; the 2009 A(H1N1)p and the 2020 COVID‐19 pandemic. Our study exploits surveillance data from the FluNet, a publicly open database operated by the WHO. To our knowledge, this is the first systematic analysis of the duration of influenza epidemics at global level using the WHO‐FluNet database, and results of this analysis deserve attention. The typical duration of the influenza epidemics was shorter than 15 weeks (median 9 weeks) for all countries lying at a latitude above 30°N and below 26°S (except China). Within this range of latitudes, influenza epidemics varied instead largely, from less than 10 weeks to over 35 weeks (i.e., from short epidemics to nearly year‐round viral circulation). The apparent tendency towards a longer duration of influenza epidemics with growing latitudes for countries lying above 40°N had never been reported before and came unexpected, and while it deserves attention as it may have some public health implications (because of the waning influenza vaccine effectiveness), we are unable to explain it convincingly. By and large, climatic and ecological drivers (such as temperature, humidity, and precipitations) and differences in cultural and social aspects and in countries' demographics all affect the circulation of influenza viruses and thus contribute to shape the seasonality of epidemics.^21^, ^22^, ^23^ However, much remains to be understood, especially regarding the differences observed between tropical countries located at very similar latitudes or even bordering one another.

In terms of seasonality of epidemics, the majority of influenza cases occurred, as expected, during winter months in the Southern and Northern hemispheres, while much more variability emerged in the intertropical belt. In terms of virus type circulation, type A virus accounted for the majority of cases in over five sixths of all seasons (84.7%); however, type B influenza viruses accounted for a median of 25% of all influenza cases in a season. Statistically significant differences in influenza type A and B viruses circulation existed geographically, across both latitudinal areas (highest median percentages of influenza A cases were in Southern hemisphere, and lowest in the intertropical belt) and WHO regions (highest in Americas and in the European regions, and lowest in the Africa and the South‐East Asia regions). We also observed large variability by seasons, with the median proportion of influenza A cases ranged between 60.9% in 2017 and 88.7% in 2018. The median % of influenza cases accounted for by each virus subtype and lineage was 29.5% for A(H3N2), 23.4% for A(H1N1), 13.8% for other/unsubtyped A viruses, 5.8% for B Yamagata, 3.3% for B Victoria, and 22.8% for uncharacterized B viruses. These findings are in fair accordance with the existing literature on the topic, especially for what concerns the patterns of circulation of the different virus types (A vs. B), subtypes and lineages^24^, ^25^ and the typical timing of the epidemic peak and its association with the country's latitude.^17^, ^26^, ^27^ Furthermore, our findings support the idea of the unpredictable nature of influenza.^28^ For instance, we observed a contrast between 2017, being the season with the lowest proportion of influenza A cases and the highest proportion of B (mostly Yamagata lineage), and 2018 presenting the highest proportion of influenza A and the near absence of B Yamagata (notably, this pattern persisted in subsequent seasons as well). Also, we did not note any clear trend in the relative proportion of A(H1N1) and A(H3N2), nor any evidence that either is increasing or declining.

Our findings have important implications for public health strategies related to influenza vaccination. Monitoring circulating influenza strains is needed to decide on the best composition for next season vaccines (also considering that viral strains differ in their ability to infect people of different ages and thus in the burden of infections and deaths that can cause),^29^, ^30^ and understanding the temporal characteristics of influenza epidemics is essential for planning the timing of influenza vaccination. In this regard, our findings suggest that in countries where influenza epidemics are typically shorter than 15 weeks (i.e., all those lying north of 30°N and south of 26°S except China, plus several of those lying at intermediate latitudes) the timing of immunization campaigns is absolutely key, because a well‐timed vaccination is likely to cover most of the influenza season, considering that vaccine‐induced protection appears to remain at fairly good levels for about 3–4 months upon vaccination.^9^ On the contrary, the optimal timing for vaccination may be less clear in the intertropical belt countries characterized by a lack of seasonality. Therefore, the recommendation may be to simply use the most recent vaccine formulation recommended by the WHO at any time of the year.^7^ The data we present here are of even greater importance given the huge impact that the COVID‐19 pandemic is having on the circulation of influenza viruses. Major reductions in influenza activity have been observed globally since shortly after the pandemic began (i.e., mid‐2020 to late 2020), with only minor outbreaks detected in some tropical regions^14^ and, to date, we do not have sufficient data to conclude if the relative proportions of circulating strains changed. This dramatic reduction may have ensued from the non‐pharmaceutical interventions (NPIs) imposed by governments worldwide in an attempt to tackle the spread of the pandemic (e.g., personal hygiene measures like face masks and restrictions in population mixing and travels) and reduction of international travel, but viral interference may also have played a role,^14^ although more research is needed in this area. Continued syndromic and virological surveillance, timely analysis of newly collected data and comparison with pre‐pandemic data, are all critically important for capturing any changes in global influenza epidemiology and gain an understanding of new influenza dynamics that could bring huge benefits to the effectiveness of vaccination programs.

Our analysis included over 4.6 million influenza cases that occurred in 149 countries during 11 consecutive seasons. This is a very large amount of data that ensures considerable robustness to our findings, yet some limitations need to be acknowledged. Data presented in our study are from surveillance systems and therefore do not provide an estimate of actual influenza burden (e.g., incidence, mortality, or hospitalization rates), but only information on the relative proportion of circulating strains. The surveillance systems of respiratory viruses are run differently in different countries and can change over time within the same country. The uneven distribution of influenza cases among countries and seasons to which this contributes (which is also partly caused by the obvious differences across countries in terms of population size and age structure) may reduce the comparability of data across countries and seasons. Moreover, the large number of unsubtyped A and uncharacterized B type influenza cases limited the power to discriminate which seasons were dominated by a particular subtype or lineage. In this regard, it is useful to point out that the large proportion (>30%) of unsubtyped A cases in the study database was driven by the fact that approximately half of influenza A cases reported in the United States (which contributed nearly 30% of all cases in the database) were unsubtyped. On the other hand, most countries rarely characterized B type influenza cases, creating a disproportion between characterized and uncharacterized B cases. Another limitation of our study is having assigned large countries to a particular latitudinal area depending on their centroids. Large countries that span many degrees in latitude (like China, Brazil, and India) can encompass regions with very diverse climate types, and the lack of geographically stratified data may lead to inaccurate results. Finally, FluNet database contains only aggregated data. Future research may focus on analysing influenza circulation among age groups or antigenic properties of the influenza viruses from ferret and human serology data.

In conclusion, this work provides an important baseline reference to better understand factors that influence seasonal influenza dynamics worldwide. Future research should continue to expand our knowledge regarding the global dynamics of influenza infections and aim to understand whether and how the emergence of the SARS‐CoV‐2 has changed the patterns of circulation of influenza viruses and the intensity and timing of influenza epidemics. An improved understanding of these aspects may greatly help policy makers in their effort to contain the burden of disease of influenza globally.

## AUTHOR CONTRIBUTIONS

**Saverio Caini:** Conceptualization; data curation; formal analysis; investigation; methodology; supervision; visualization. **Guglielmo Bonaccorsi:** Conceptualization; funding acquisition; methodology; project administration; resources; supervision. **Chiara Lorini:** Funding acquisition; investigation; project administration; resources; supervision; validation. **Mendel Haag:** Conceptualization; methodology; project administration; resources. **Ian McGovern:** Conceptualization; project administration; resources; validation. **W. John Paget:** Conceptualization; investigation; methodology; software; supervision; validation. **Patrizio Zanobini:** Data curation; formal analysis; investigation; methodology; software; visualization.

## CONFLICT OF INTEREST

Mendel Haag and Ian McGovern are employees of Seqirus. JP declares that Nivel has received influenza research grants from the World Health Organization, Sanofi Pasteur, and the Foundation for Influenza Epidemiology. The other Authors declare no conflicts of interest relevant to this article.

### PEER REVIEW

The peer review history for this article is available at https://publons.com/publon/10.1111/irv.12969.

## Supporting information


**Table S1.** Circulation of type A and B influenza viruses by country. WHO FluNet database 2010–2020. Only seasons with ≥ 50 reported influenza cases were included in the analysis. See text for details.
**Table S2**. Circulation of type A and B influenza viruses by season and latitudinal area. WHO FluNet database 2010–2020. Only seasons with ≥50 reported influenza cases overall were included in the analysis. See text for details.
**Table S3**. Circulation of the different influenza virus type A subtypes and type B lineages by country. WHO FluNet database 2010–2020. Only seasons with ≥50 reported influenza cases overall were included in the analysis. See text for details.
**Table S4**. Typical timing and amplitude of the primary and secondary peak, and median duration (in weeks), of influenza epidemics by country (sorted according to the latitude of the country centroid). WHO FluNet database 2010–2019. Only countries with ≥ 5 seasons with ≥ 50 reported influenza cases were included in the analysis. See text for details.

## Data Availability

The data are publicly available and can be downloaded from the WHO FluNet website.
