# Supplementary material for: Global patterns of seasonal influenza activity, duration of activity and virus (sub)type circulation from 2010 to 2020
Source: Influenza Other Respir Viruses. 2022 Feb 24;16(4):696–706. doi: 10.1111/irv.12969 (PMC9178051; doi:10.1111/irv.12969)
Supplement: Supplementary file 1 — Table S1. Circulation of type A and B influenza viruses by country. WHO FluNet database 2010–2020. Only seasons with ≥ 50 reported influenza cases were included in the analysis. See text for details. Table S2. Circulation of type A and B influenza viruses by season and latitudinal area. WHO FluNet database 2010–2020. Only seasons with ≥50 reported influenza cases overall were included in the analysis. See text for details. Table S3. Circulation of the different influenza virus type A subtypes and type B lineages by country. WHO FluNet database 2010–2020. Only seasons with ≥50 reported influenza cases overall were included in the analysis. See text for details. Table S4. Typical timing and amplitude of the primary and secondary peak, and median duration (in weeks), of influenza epidemics by country (sorted according to the latitude of the country centroid). WHO FluNet database 2010–2019. Only countries with ≥ 5 seasons with ≥ 50 reported influenza cases were included in the analysis. See text for details. [file IRV-16-696-s001.docx]

**Table S1**. Circulation of type A and B influenza viruses by country. WHO FluNet database 2010-2020. Only seasons with ≥ 50 reported influenza cases were included in the analysis. See text for details.

| **Country** | **N season (≥ 50 cases)** | **N influenza cases** | **Influenza type A** | | **Influenza type B** | | **Median cases per season** | **Median % A** | **Country Seasons with % A** | | | |
| --- | --- | --- | --- | --- | --- | --- | --- | --- | --- | --- | --- | --- |
|  |  |  | **N** | **%** | **N** | **%** |  |  | **≥ 80%** | **≥ 50% to < 80%** | **≥ 20% to < 50%** | **< 20%** |
| Afghanistan | 4 | 896 | 744 | 83.0% | 152 | 17.0% | 225 | 80% | 2 | 2 | 0 | 0 |
| Albania | 10 | 2,358 | 1,714 | 72.7% | 644 | 27.3% | 185 | 75% | 5 | 3 | 2 | 0 |
| Algeria | 9 | 2,489 | 1,671 | 67.1% | 818 | 32.9% | 195 | 71% | 3 | 4 | 2 | 0 |
| Argentina | 11 | 43,910 | 36,107 | 82.2% | 7,803 | 17.8% | 3.039 | 84% | 6 | 5 | 0 | 0 |
| Armenia | 6 | 2,175 | 1,602 | 73.7% | 573 | 26.3% | 377 | 53% | 2 | 1 | 3 | 0 |
| Aruba | 4 | 381 | 318 | 83.5% | 63 | 16.5% | 82 | 85% | 3 | 1 | 0 | 0 |
| Australia | 11 | 53,619 | 42,275 | 78.8% | 11,344 | 21.2% | 3.625 | 82% | 7 | 4 | 0 | 0 |
| Austria | 10 | 26,956 | 18,951 | 70.3% | 8,005 | 29.7% | 2.437 | 78% | 4 | 4 | 2 | 0 |
| Azerbaijan | 5 | 860 | 416 | 48.4% | 444 | 51.6% | 140 | 45% | 2 | 0 | 2 | 1 |
| Bahrain | 8 | 1,589 | 1,251 | 78.7% | 338 | 21.3% | 161 | 74% | 2 | 6 | 0 | 0 |
| Bangladesh | 10 | 7,878 | 5,138 | 65.2% | 2,740 | 34.8% | 679 | 67% | 1 | 9 | 0 | 0 |
| Barbados | 2 | 128 | 80 | 62.5% | 48 | 37.5% | 64 | 61% | 1 | 0 | 1 | 0 |
| Belarus | 10 | 6,167 | 5,181 | 84.0% | 986 | 16.0% | 503 | 83% | 6 | 4 | 0 | 0 |
| Belgium | 10 | 4,369 | 2,992 | 68.5% | 1,377 | 31.5% | 354 | 84% | 6 | 1 | 3 | 0 |
| Belize | 1 | 53 | 24 | 45.3% | 29 | 54.7% | 53 | 45% | 0 | 0 | 1 | 0 |
| Bhutan | 9 | 2,837 | 1,852 | 65.3% | 985 | 34.7% | 253 | 59% | 0 | 7 | 2 | 0 |
| Bolivia | 11 | 11,110 | 8,839 | 79.6% | 2,271 | 20.4% | 936 | 82% | 6 | 5 | 0 | 0 |
| Bosnia and Herzegovina | 3 | 759 | 721 | 95.0% | 38 | 5.0% | 110 | 91% | 2 | 1 | 0 | 0 |
| Brazil | 11 | 34,800 | 27,158 | 78.0% | 7,642 | 22.0% | 1.744 | 77% | 5 | 6 | 0 | 0 |
| Bulgaria | 10 | 3,552 | 2,550 | 71.8% | 1,002 | 28.2% | 327 | 78% | 5 | 2 | 1 | 2 |
| Burkina Faso | 6 | 658 | 494 | 75.1% | 164 | 24.9% | 104 | 77% | 1 | 5 | 0 | 0 |
| Cambodia | 11 | 4,799 | 3,064 | 63.8% | 1,735 | 36.2% | 276 | 76% | 5 | 4 | 2 | 0 |
| Cameroon | 11 | 4,058 | 2,558 | 63.0% | 1,500 | 37.0% | 414 | 74% | 3 | 5 | 3 | 0 |
| Canada | 10 | 378,446 | 279,714 | 73.9% | 98,732 | 26.1% | 39.411 | 77% | 5 | 4 | 1 | 0 |
| Central African Republic | 6 | 802 | 503 | 62.7% | 299 | 37.3% | 143 | 63% | 3 | 0 | 2 | 1 |
| Chile | 11 | 32,698 | 24,769 | 75.8% | 7,929 | 24.2% | 2.600 | 77% | 4 | 7 | 0 | 0 |
| China | 10 | 678,837 | 466,889 | 68.8% | 211,948 | 31.2% | 66.032 | 73% | 3 | 6 | 1 | 0 |
| Colombia | 11 | 8,602 | 7,375 | 85.7% | 1,227 | 14.3% | 695 | 90% | 8 | 3 | 0 | 0 |
| Congo, Democratic Republic of | 10 | 1,530 | 976 | 63.8% | 554 | 36.2% | 156 | 63% | 2 | 7 | 1 | 0 |
| Costa Rica | 11 | 5,108 | 4,137 | 81.0% | 971 | 19.0% | 459 | 85% | 7 | 4 | 0 | 0 |
| Croatia | 10 | 17,042 | 14,104 | 82.8% | 2,938 | 17.2% | 1.413 | 91% | 7 | 2 | 1 | 0 |
| Cuba | 11 | 5,252 | 4,186 | 79.7% | 1,066 | 20.3% | 357 | 83% | 6 | 3 | 2 | 0 |
| Cyprus | 4 | 1,037 | 873 | 84.2% | 164 | 15.8% | 146 | 76% | 1 | 2 | 1 | 0 |
| Czechia | 9 | 4,453 | 3,214 | 72.2% | 1,239 | 27.8% | 429 | 77% | 4 | 4 | 1 | 0 |
| Denmark | 10 | 54,311 | 36,015 | 66.3% | 18,296 | 33.7% | 4.159 | 73% | 4 | 4 | 2 | 0 |
| Dominican Republic | 10 | 2,015 | 1,465 | 72.7% | 550 | 27.3% | 105 | 79% | 5 | 4 | 1 | 0 |
| Ecuador | 11 | 6,187 | 5,413 | 87.5% | 774 | 12.5% | 505 | 89% | 8 | 3 | 0 | 0 |
| Egypt | 10 | 15,680 | 11,736 | 74.8% | 3,944 | 25.2% | 1.813 | 70% | 2 | 8 | 0 | 0 |
| El Salvador | 10 | 2,300 | 1,631 | 70.9% | 669 | 29.1% | 235 | 73% | 4 | 4 | 2 | 0 |
| Estonia | 10 | 11,031 | 8,236 | 74.7% | 2,795 | 25.3% | 764 | 80% | 5 | 4 | 1 | 0 |
| Ethiopia | 7 | 1,273 | 904 | 71.0% | 369 | 29.0% | 203 | 63% | 2 | 5 | 0 | 0 |
| Fiji | 6 | 679 | 389 | 57.3% | 290 | 42.7% | 110 | 69% | 2 | 2 | 1 | 1 |
| Finland | 10 | 2,148 | 1,638 | 76.3% | 510 | 23.7% | 172 | 86% | 7 | 2 | 1 | 0 |
| France | 10 | 183,788 | 129,882 | 70.7% | 53,906 | 29.3% | 19.657 | 71% | 4 | 4 | 2 | 0 |
| French Guiana | 10 | 1,625 | 1,090 | 67.1% | 535 | 32.9% | 162 | 77% | 5 | 3 | 2 | 0 |
| Georgia | 9 | 2,385 | 1,629 | 68.3% | 756 | 31.7% | 173 | 69% | 4 | 3 | 1 | 1 |
| Germany | 10 | 14,067 | 9,642 | 68.5% | 4,425 | 31.5% | 1.472 | 80% | 5 | 3 | 2 | 0 |
| Ghana | 11 | 6,212 | 4,533 | 73.0% | 1,679 | 27.0% | 469 | 63% | 4 | 5 | 2 | 0 |
| Greece | 10 | 12,792 | 11,050 | 86.4% | 1,742 | 13.6% | 810 | 90% | 6 | 1 | 3 | 0 |
| Guadeloupe | 6 | 567 | 481 | 84.8% | 86 | 15.2% | 88 | 84% | 5 | 1 | 0 | 0 |
| Guatemala | 11 | 2,233 | 1,766 | 79.1% | 467 | 20.9% | 210 | 80% | 6 | 4 | 1 | 0 |
| Guinea | 2 | 198 | 118 | 59.6% | 80 | 40.4% | 99 | 55% | 0 | 1 | 1 | 0 |
| Haiti | 5 | 953 | 789 | 82.8% | 164 | 17.2% | 111 | 88% | 3 | 0 | 2 | 0 |
| Honduras | 10 | 2,101 | 1,527 | 72.7% | 574 | 27.3% | 195 | 76% | 4 | 4 | 2 | 0 |
| Hungary | 10 | 5,919 | 4,233 | 71.5% | 1,686 | 28.5% | 590 | 78% | 5 | 2 | 3 | 0 |
| Iceland | 10 | 3,323 | 2,388 | 71.9% | 935 | 28.1% | 295 | 72% | 4 | 4 | 1 | 1 |
| India | 11 | 34,068 | 29,491 | 86.6% | 4,577 | 13.4% | 1.930 | 87% | 7 | 4 | 0 | 0 |
| Indonesia | 11 | 9,384 | 5,646 | 60.2% | 3,738 | 39.8% | 878 | 60% | 1 | 9 | 1 | 0 |
| Iran | 10 | 19,073 | 15,350 | 80.5% | 3,723 | 19.5% | 1.114 | 75% | 5 | 4 | 1 | 0 |
| Iraq | 8 | 2,813 | 2,399 | 85.3% | 414 | 14.7% | 297 | 91% | 5 | 3 | 0 | 0 |
| Ireland | 10 | 23,065 | 16,397 | 71.1% | 6,668 | 28.9% | 2.015 | 74% | 4 | 5 | 1 | 0 |
| Israel | 10 | 13,522 | 9,926 | 73.4% | 3,596 | 26.6% | 1.386 | 74% | 4 | 5 | 1 | 0 |
| Italy | 10 | 30,559 | 22,665 | 74.2% | 7,894 | 25.8% | 2.851 | 82% | 5 | 2 | 3 | 0 |
| Ivory Coast | 11 | 3,385 | 2,112 | 62.4% | 1,273 | 37.6% | 287 | 63% | 2 | 6 | 2 | 1 |
| Jamaica | 8 | 1,209 | 948 | 78.4% | 261 | 21.6% | 95 | 84% | 4 | 2 | 1 | 1 |
| Japan | 10 | 82,589 | 62,252 | 75.4% | 20,337 | 24.6% | 7.895 | 80% | 5 | 5 | 0 | 0 |
| Jordan | 10 | 2,640 | 2,176 | 82.4% | 464 | 17.6% | 184 | 90% | 8 | 0 | 2 | 0 |
| Kazakhstan | 10 | 6,079 | 4,622 | 76.0% | 1,457 | 24.0% | 576 | 73% | 4 | 6 | 0 | 0 |
| Kenya | 9 | 3,671 | 2,588 | 70.5% | 1,083 | 29.5% | 220 | 73% | 4 | 5 | 0 | 0 |
| Kosovo | 4 | 1,320 | 827 | 62.7% | 493 | 37.3% | 314 | 45% | 1 | 0 | 3 | 0 |
| Kuwait | 3 | 13,104 | 10,150 | 77.5% | 2,954 | 22.5% | 5.509 | 79% | 1 | 2 | 0 | 0 |
| Kyrgyzstan | 8 | 1,481 | 831 | 56.1% | 650 | 43.9% | 141 | 43% | 1 | 2 | 4 | 1 |
| Laos | 10 | 4,596 | 2,869 | 62.4% | 1,727 | 37.6% | 499 | 66% | 1 | 7 | 2 | 0 |
| Latvia | 10 | 19,746 | 13,748 | 69.6% | 5,998 | 30.4% | 2.200 | 71% | 4 | 5 | 1 | 0 |
| Lebanon | 6 | 1,155 | 637 | 55.2% | 518 | 44.8% | 170 | 55% | 1 | 2 | 3 | 0 |
| Lithuania | 10 | 7,302 | 5,677 | 77.7% | 1,625 | 22.3% | 769 | 81% | 6 | 3 | 0 | 1 |
| Luxembourg | 10 | 4,518 | 2,781 | 61.6% | 1,737 | 38.4% | 412 | 66% | 4 | 3 | 3 | 0 |
| Madagascar | 10 | 4,700 | 2,724 | 58.0% | 1,976 | 42.0% | 473 | 55% | 1 | 6 | 3 | 0 |
| Malaysia | 9 | 3,297 | 2,097 | 63.6% | 1,200 | 36.4% | 292 | 73% | 4 | 4 | 1 | 0 |
| Maldives | 3 | 910 | 701 | 77.0% | 209 | 23.0% | 378 | 84% | 2 | 0 | 0 | 1 |
| Mali | 9 | 1,182 | 758 | 64.1% | 424 | 35.9% | 144 | 59% | 3 | 3 | 3 | 0 |
| Malta | 8 | 3,710 | 2,546 | 68.6% | 1,164 | 31.4% | 396 | 79% | 4 | 3 | 1 | 0 |
| Martinique | 6 | 994 | 742 | 74.6% | 252 | 25.4% | 177 | 71% | 2 | 3 | 1 | 0 |
| Mauritania | 1 | 51 | 44 | 86.3% | 7 | 13.7% | 51 | 86% | 1 | 0 | 0 | 0 |
| Mauritius | 10 | 1,369 | 1,180 | 86.2% | 189 | 13.8% | 105 | 91% | 6 | 3 | 1 | 0 |
| Mexico | 10 | 57,154 | 46,033 | 80.5% | 11,121 | 19.5% | 5.517 | 77% | 4 | 6 | 0 | 0 |
| Moldova | 9 | 2,493 | 1,819 | 73.0% | 674 | 27.0% | 225 | 72% | 3 | 5 | 0 | 1 |
| Mongolia | 10 | 5,692 | 4,310 | 75.7% | 1,382 | 24.3% | 477 | 82% | 5 | 3 | 2 | 0 |
| Montenegro | 5 | 1,588 | 1,192 | 75.1% | 396 | 24.9% | 267 | 94% | 3 | 1 | 0 | 1 |
| Morocco | 9 | 2,898 | 2,053 | 70.8% | 845 | 29.2% | 271 | 60% | 3 | 3 | 3 | 0 |
| Mozambique | 3 | 235 | 149 | 63.4% | 86 | 36.6% | 78 | 55% | 1 | 2 | 0 | 0 |
| Myanmar | 4 | 1,740 | 1,289 | 74.1% | 451 | 25.9% | 416 | 77% | 2 | 1 | 1 | 0 |
| Nepal | 9 | 12,122 | 8,552 | 70.5% | 3,570 | 29.5% | 1.122 | 68% | 3 | 3 | 2 | 1 |
| Netherlands | 10 | 26,509 | 21,202 | 80.0% | 5,307 | 20.0% | 3.053 | 86% | 6 | 3 | 1 | 0 |
| New Caledonia | 10 | 2,162 | 1,462 | 67.6% | 700 | 32.4% | 191 | 71% | 4 | 2 | 2 | 2 |
| New Zealand | 10 | 17,235 | 11,533 | 66.9% | 5,702 | 33.1% | 1.115 | 74% | 5 | 3 | 2 | 0 |
| Nicaragua | 10 | 7,975 | 5,528 | 69.3% | 2,447 | 30.7% | 811 | 70% | 3 | 4 | 3 | 0 |
| Niger | 7 | 710 | 479 | 67.5% | 231 | 32.5% | 114 | 77% | 3 | 3 | 1 | 0 |
| Nigeria | 9 | 1,241 | 806 | 64.9% | 435 | 35.1% | 115 | 74% | 1 | 7 | 1 | 0 |
| North Korea | 4 | 973 | 882 | 90.6% | 91 | 9.4% | 205 | 86% | 3 | 1 | 0 | 0 |
| North Macedonia | 3 | 944 | 713 | 75.5% | 231 | 24.5% | 271 | 59% | 1 | 2 | 0 | 0 |
| Norway | 10 | 152,992 | 100,988 | 66.0% | 52,004 | 34.0% | 15.374 | 65% | 4 | 4 | 2 | 0 |
| Oman | 11 | 11,316 | 8,396 | 74.2% | 2,920 | 25.8% | 1.003 | 76% | 3 | 8 | 0 | 0 |
| Pakistan | 9 | 3,718 | 3,220 | 86.6% | 498 | 13.4% | 442 | 85% | 6 | 3 | 0 | 0 |
| Panama | 10 | 2,544 | 2,097 | 82.4% | 447 | 17.6% | 204 | 91% | 6 | 1 | 3 | 0 |
| Papua New Guinea | 1 | 144 | 117 | 81.3% | 27 | 18.8% | 144 | 81% | 1 | 0 | 0 | 0 |
| Paraguay | 11 | 9,206 | 7,075 | 76.9% | 2,131 | 23.1% | 932 | 72% | 4 | 6 | 0 | 1 |
| Peru | 11 | 7,688 | 6,202 | 80.7% | 1,486 | 19.3% | 391 | 75% | 5 | 6 | 0 | 0 |
| Philippines | 10 | 6,018 | 3,370 | 56.0% | 2,648 | 44.0% | 434 | 60% | 1 | 7 | 2 | 0 |
| Poland | 9 | 14,404 | 10,968 | 76.1% | 3,436 | 23.9% | 1.700 | 87% | 6 | 2 | 1 | 0 |
| Portugal | 10 | 22,620 | 16,961 | 75.0% | 5,659 | 25.0% | 1.614 | 76% | 5 | 3 | 2 | 0 |
| Qatar | 10 | 44,488 | 33,026 | 74.2% | 11,462 | 25.8% | 3.504 | 76% | 2 | 8 | 0 | 0 |
| Romania | 10 | 11,799 | 7,961 | 67.5% | 3,838 | 32.5% | 1.300 | 61% | 4 | 3 | 3 | 0 |
| Russia | 10 | 183,366 | 141,740 | 77.3% | 41,626 | 22.7% | 17.811 | 77% | 4 | 6 | 0 | 0 |
| Rwanda | 7 | 722 | 610 | 84.5% | 112 | 15.5% | 88 | 86% | 5 | 2 | 0 | 0 |
| Saudi Arabia | 4 | 4,947 | 3,634 | 73.5% | 1,313 | 26.5% | 1.450 | 72% | 0 | 4 | 0 | 0 |
| Senegal | 11 | 7,283 | 4,715 | 64.7% | 2,568 | 35.3% | 483 | 61% | 3 | 5 | 3 | 0 |
| Serbia | 10 | 6,275 | 4,909 | 78.2% | 1,366 | 21.8% | 446 | 85% | 7 | 2 | 1 | 0 |
| Sierra Leone | 2 | 138 | 104 | 75.4% | 34 | 24.6% | 69 | 78% | 1 | 1 | 0 | 0 |
| Singapore | 11 | 12,953 | 9,259 | 71.5% | 3,694 | 28.5% | 998 | 69% | 2 | 9 | 0 | 0 |
| Slovakia | 10 | 3,398 | 2,169 | 63.8% | 1,229 | 36.2% | 308 | 81% | 5 | 2 | 2 | 1 |
| Slovenia | 10 | 25,722 | 18,452 | 71.7% | 7,270 | 28.3% | 2.616 | 67% | 4 | 6 | 0 | 0 |
| South Africa | 11 | 11,903 | 8,510 | 71.5% | 3,393 | 28.5% | 1.164 | 80% | 6 | 4 | 1 | 0 |
| South Korea | 10 | 18,417 | 12,062 | 65.5% | 6,355 | 34.5% | 1.763 | 63% | 3 | 4 | 3 | 0 |
| Spain | 10 | 103,902 | 77,174 | 74.3% | 26,728 | 25.7% | 8.947 | 72% | 4 | 4 | 2 | 0 |
| Sri Lanka | 11 | 9,839 | 7,231 | 73.5% | 2,608 | 26.5% | 812 | 69% | 4 | 5 | 2 | 0 |
| Suriname | 3 | 291 | 265 | 91.1% | 26 | 8.9% | 65 | 88% | 2 | 1 | 0 | 0 |
| Sweden | 10 | 98,447 | 69,124 | 70.2% | 29,323 | 29.8% | 9.118 | 70% | 4 | 4 | 2 | 0 |
| Switzerland | 10 | 65,414 | 47,376 | 72.4% | 18,038 | 27.6% | 4.912 | 71% | 3 | 5 | 2 | 0 |
| Tajikistan | 1 | 62 | 9 | 14.5% | 53 | 85.5% | 62 | 15% | 0 | 0 | 0 | 1 |
| Tanzania | 11 | 2,302 | 1,604 | 69.7% | 698 | 30.3% | 227 | 70% | 0 | 11 | 0 | 0 |
| Thailand | 11 | 10,509 | 6,875 | 65.4% | 3,634 | 34.6% | 908 | 67% | 3 | 6 | 2 | 0 |
| Timor-Leste | 2 | 372 | 181 | 48.7% | 191 | 51.3% | 186 | 45% | 0 | 1 | 1 | 0 |
| Togo | 10 | 1,752 | 1,044 | 59.6% | 708 | 40.4% | 125 | 59% | 2 | 5 | 3 | 0 |
| Trinidad and Tobago | 1 | 270 | 270 | 100.0% | 0 | 0.0% | 270 | 100% | 1 | 0 | 0 | 0 |
| Tunisia | 10 | 2,647 | 2,108 | 79.6% | 539 | 20.4% | 245 | 81% | 5 | 5 | 0 | 0 |
| Turkey | 10 | 25,219 | 18,721 | 74.2% | 6,498 | 25.8% | 1.956 | 72% | 3 | 5 | 2 | 0 |
| Uganda | 9 | 3,100 | 2,213 | 71.4% | 887 | 28.6% | 311 | 76% | 4 | 4 | 1 | 0 |
| UK | 10 | 176,557 | 132,787 | 75.2% | 43,770 | 24.8% | 13.923 | 80% | 5 | 5 | 0 | 0 |
| Ukraine | 10 | 16,543 | 11,699 | 70.7% | 4,844 | 29.3% | 1.279 | 88% | 6 | 2 | 1 | 1 |
| United Arab Emirates | 1 | 183 | 111 | 60.7% | 72 | 39.3% | 183 | 61% | 0 | 1 | 0 | 0 |
| Uruguay | 9 | 1,290 | 1,123 | 87.1% | 167 | 12.9% | 124 | 89% | 7 | 2 | 0 | 0 |
| USA | 10 | 1,437,794 | 1,039,306 | 72.3% | 398,488 | 27.7% | 113.867 | 73% | 4 | 6 | 0 | 0 |
| Uzbekistan | 4 | 387 | 233 | 60.2% | 154 | 39.8% | 89 | 62% | 1 | 2 | 1 | 0 |
| Venezuela | 6 | 3,698 | 3,570 | 96.5% | 128 | 3.5% | 95 | 78% | 3 | 2 | 1 | 0 |
| Viet Nam | 11 | 7,230 | 4,727 | 65.4% | 2,503 | 34.6% | 715 | 70% | 3 | 5 | 3 | 0 |
| West Bank and Gaza Strip | 3 | 1,888 | 1,789 | 94.8% | 99 | 5.2% | 543 | 91% | 3 | 0 | 0 | 0 |
| Yemen | 2 | 315 | 294 | 93.3% | 21 | 6.7% | 158 | 93% | 2 | 0 | 0 | 0 |
| Zambia | 10 | 1,771 | 939 | 53.0% | 832 | 47.0% | 146 | 56% | 0 | 6 | 4 | 0 |
| **TOTAL** | **1.244** | **4,659,001** | **3,379,199** | **72.5%** | **1,279,802** | **27.5%** | **494** | **75%** | **527**  **(42.3%)** | **526**  **(42.3%)** | **170**  **(13.7%)** | **21**  **(1.7%)** |

**Table S2**. Circulation of type A and B influenza viruses by season and latitudinal area. WHO FluNet database 2010-2020. Only seasons with ≥50 reported influenza cases overall were included in the analysis. See text for details.

| **Geographical area** | **Season** | **N country seasons (≥50 cases)** | **Influenza** | **Influenza type A** | | **Influenza type B** | | **Median cases per season** | **Median % A** | **Country Seasons with % A** | | | |
| --- | --- | --- | --- | --- | --- | --- | --- | --- | --- | --- | --- | --- | --- |
|  |  |  | **N total** | **N** | **%** | **N** | **%** |  |  | **≥ 80%** | **≥50% to <80%** | **≥20% to <50%** | **< 20%** |
| Northern hemisphere | 2010 | 59 | 243,264 | 182,385 | 75.0% | 60,879 | 25.0% | 931 | 72.8% | 21 | 28 | 7 | 3 |
|  | 2011 | 55 | 150,352 | 103,804 | 69.0% | 46,548 | 31.0% | 510 | 88.3% | 36 | 12 | 6 | 1 |
|  | 2012 | 63 | 270,972 | 190,655 | 70.4% | 80,317 | 29.6% | 838 | 64.9% | 13 | 37 | 13 | 0 |
|  | 2013 | 58 | 240,833 | 191,667 | 79.6% | 49,166 | 20.4% | 663 | 92.5% | 42 | 15 | 1 | 0 |
|  | 2014 | 64 | 395,159 | 301,391 | 76.3% | 93,768 | 23.7% | 817 | 72.5% | 15 | 39 | 9 | 1 |
|  | 2015 | 69 | 409,257 | 270,716 | 66.1% | 138,541 | 33.9% | 991 | 68.7% | 26 | 29 | 14 | 0 |
|  | 2016 | 67 | 458,211 | 371,104 | 81.0% | 87,107 | 19.0% | 878 | 86.3% | 42 | 21 | 4 | 0 |
|  | 2017 | 73 | 780,821 | 451,368 | 57.8% | 329,453 | 42.2% | 926 | 46.7% | 7 | 26 | 32 | 8 |
|  | 2018 | 72 | 641,268 | 590,543 | 92.1% | 50,725 | 7.9% | 1,054 | 99.0% | 62 | 10 | 0 | 0 |
|  | 2019 | 73 | 622,245 | 388,660 | 62.5% | 233,585 | 37.5% | 837 | 71.6% | 23 | 43 | 7 | 0 |
| Inter-tropical belt | 2010 | 44 | 32,543 | 26,517 | 81.5% | 6,026 | 18.5% | 460 | 83.3% | 27 | 13 | 3 | 1 |
|  | 2011 | 43 | 24,751 | 18,412 | 74.4% | 6,339 | 25.6% | 368 | 70.9% | 18 | 15 | 10 | 0 |
|  | 2012 | 46 | 22,667 | 14,674 | 64.7% | 7,993 | 35.3% | 345 | 64.2% | 13 | 24 | 9 | 0 |
|  | 2013 | 46 | 27,857 | 20,213 | 72.6% | 7,644 | 27.4% | 279 | 71.3% | 14 | 24 | 8 | 0 |
|  | 2014 | 44 | 18,064 | 11,992 | 66.4% | 6,072 | 33.6% | 256 | 62.9% | 12 | 24 | 8 | 0 |
|  | 2015 | 42 | 21,699 | 17,739 | 81.7% | 3,960 | 18.3% | 267 | 79.9% | 21 | 17 | 2 | 2 |
|  | 2016 | 56 | 26,957 | 19,644 | 72.9% | 7,313 | 27.1% | 233 | 75.7% | 27 | 16 | 13 | 0 |
|  | 2017 | 52 | 31,764 | 22,821 | 71.8% | 8,943 | 28.2% | 278 | 69.8% | 16 | 27 | 7 | 2 |
|  | 2018 | 55 | 31,668 | 24,383 | 77.0% | 7,285 | 23.0% | 262 | 70.4% | 16 | 33 | 5 | 1 |
|  | 2019 | 61 | 40,606 | 30,810 | 75.9% | 9,796 | 24.1% | 346 | 76.0% | 23 | 33 | 4 | 1 |
|  | 2020 | 39 | 7,388 | 5,384 | 72.9% | 2,004 | 27.1% | 122 | 74.7% | 18 | 15 | 5 | 1 |
| Southern hemisphere | 2010 | 6 | 8,136 | 5,644 | 69.4% | 2,492 | 30.6% | 1,293 | 74.6% | 3 | 2 | 1 | 0 |
|  | 2011 | 6 | 8,448 | 7,146 | 84.6% | 1,302 | 15.4% | 1,365 | 90.0% | 5 | 1 | 0 | 0 |
|  | 2012 | 6 | 13,548 | 9,439 | 69.7% | 4,109 | 30.3% | 2,172 | 66.0% | 1 | 5 | 0 | 0 |
|  | 2013 | 6 | 15,108 | 12,515 | 82.8% | 2,593 | 17.2% | 2,094 | 83.5% | 4 | 2 | 0 | 0 |
|  | 2014 | 6 | 12,019 | 10,216 | 85.0% | 1,803 | 15.0% | 2,116 | 84.1% | 5 | 1 | 0 | 0 |
|  | 2015 | 5 | 14,135 | 8,652 | 61.2% | 5,483 | 38.8% | 2,289 | 74.8% | 2 | 2 | 1 | 0 |
|  | 2016 | 6 | 18,852 | 15,546 | 82.5% | 3,306 | 17.5% | 2,355 | 85.8% | 4 | 2 | 0 | 0 |
|  | 2017 | 6 | 23,647 | 17,978 | 76.0% | 5,669 | 24.0% | 2,715 | 73.3% | 1 | 5 | 0 | 0 |
|  | 2018 | 6 | 14,830 | 11,488 | 77.5% | 3,342 | 22.5% | 2,676 | 82.5% | 4 | 2 | 0 | 0 |
|  | 2019 | 6 | 30,165 | 24,243 | 80.4% | 5,922 | 19.6% | 3,821 | 87.6% | 4 | 1 | 1 | 0 |
|  | 2020 | 4 | 1,767 | 1,450 | 82.1% | 317 | 17.9% | 331 | 80.8% | 2 | 2 | 0 | 0 |
| **Total** |  | **1,244** | **4,659,001** | **3,379,199** | **72.5%** | **1,279,802** | **27.5%** | **494** | **75.0%** | **527**  **(42.3%)** | **526**  **(42.3%)** | **170**  **(13.7%)** | **21**  **(1.7%)** |

**Table S3**. Circulation of the different influenza virus type A subtypes and type B lineages by country. WHO FluNet database 2010-2020. Only seasons with ≥50 reported influenza cases overall were included in the analysis. See text for details.

| **Country** | **N country season (≥50 cases)** | **N influenza cases** | **A(H3N2)** | | **A(H1N1)** | | **A other or unsubtyped** | | **B Yamagata** | | **B Victoria** | | **B uncharacterized** | |
| --- | --- | --- | --- | --- | --- | --- | --- | --- | --- | --- | --- | --- | --- | --- |
|  |  |  | **N** | **%** | **N** | **%** | **N** | **%** | **N** | **%** | **N** | **%** | **N** | **%** |
| Afghanistan | 4 | 896 | 268 | 29.9% | 290 | 32.4% | 186 | 20.8% | 104 | 11.6% | 42 | 4.7% | 6 | 0.7% |
| Albania | 10 | 2,358 | 574 | 24.3% | 878 | 37.2% | 262 | 11.1% | 186 | 7.9% | 275 | 11.7% | 183 | 7.8% |
| Algeria | 9 | 2,489 | 613 | 24.6% | 1,056 | 42.4% | 2 | 0.1% | 87 | 3.5% | 68 | 2.7% | 663 | 26.6% |
| Argentina | 11 | 43,910 | 8,773 | 20.0% | 11,641 | 26.5% | 15,692 | 35.7% | 1,046 | 2.4% | 917 | 2.1% | 5,840 | 13.3% |
| Armenia | 6 | 2,175 | 235 | 10.8% | 1,362 | 62.6% | 5 | 0.2% | 166 | 7.6% | 139 | 6.4% | 268 | 12.3% |
| Aruba | 4 | 381 | 0 | 0.0% | 318 | 83.5% | 0 | 0.0% | 8 | 2.1% | 0 | 0.0% | 55 | 14.4% |
| Australia | 11 | 53,619 | 18,277 | 34.1% | 8,141 | 15.2% | 15,857 | 29.6% | 105 | 0.2% | 17 | 0.0% | 11,222 | 20.9% |
| Austria | 10 | 26,956 | 3,282 | 12.2% | 3,852 | 14.3% | 11,817 | 43.8% | 1 | 0.0% | 114 | 0.4% | 7,890 | 29.3% |
| Azerbaijan | 5 | 860 | 115 | 13.4% | 18 | 2.1% | 283 | 32.9% | 13 | 1.5% | 127 | 14.8% | 304 | 35.3% |
| Bahrain | 8 | 1,589 | 217 | 13.7% | 933 | 58.7% | 101 | 6.4% | 133 | 8.4% | 90 | 5.7% | 115 | 7.2% |
| Bangladesh | 10 | 7,878 | 2,583 | 32.8% | 2,536 | 32.2% | 11 | 0.1% | 573 | 7.3% | 1,204 | 15.3% | 963 | 12.2% |
| Barbados | 2 | 128 | 12 | 9.4% | 64 | 50.0% | 4 | 3.1% | 0 | 0.0% | 10 | 7.8% | 38 | 29.7% |
| Belarus | 10 | 6,167 | 1,630 | 26.4% | 3,422 | 55.5% | 129 | 2.1% | 11 | 0.2% | 48 | 0.8% | 927 | 15.0% |
| Belgium | 10 | 4,369 | 1,315 | 30.1% | 1,104 | 25.3% | 573 | 13.1% | 565 | 12.9% | 210 | 4.8% | 602 | 13.8% |
| Belize | 1 | 53 | 3 | 5.7% | 19 | 35.8% | 2 | 3.8% | 1 | 1.9% | 27 | 50.9% | 1 | 1.9% |
| Bhutan | 9 | 2,837 | 793 | 28.0% | 1,026 | 36.2% | 33 | 1.2% | 246 | 8.7% | 334 | 11.8% | 405 | 14.3% |
| Bolivia | 11 | 11,110 | 2,367 | 21.3% | 5,706 | 51.4% | 766 | 6.9% | 512 | 4.6% | 289 | 2.6% | 1,470 | 13.2% |
| Bosnia and Herzegovina | 3 | 759 | 57 | 7.5% | 521 | 68.6% | 143 | 18.8% | 0 | 0.0% | 0 | 0.0% | 38 | 5.0% |
| Brazil | 11 | 34,800 | 9,977 | 28.7% | 16,683 | 47.9% | 480 | 1.4% | 427 | 1.2% | 593 | 1.7% | 6,622 | 19.0% |
| Bulgaria | 10 | 3,552 | 1,325 | 37.3% | 1,190 | 33.5% | 35 | 1.0% | 405 | 11.4% | 209 | 5.9% | 388 | 10.9% |
| Burkina Faso | 6 | 658 | 276 | 41.9% | 204 | 31.0% | 14 | 2.1% | 21 | 3.2% | 66 | 10.0% | 77 | 11.7% |
| Cambodia | 11 | 4,799 | 1,546 | 32.2% | 1,472 | 30.7% | 46 | 1.0% | 170 | 3.5% | 287 | 6.0% | 1,278 | 26.6% |
| Cameroon | 11 | 4,058 | 1,377 | 33.9% | 1,014 | 25.0% | 167 | 4.1% | 47 | 1.2% | 184 | 4.5% | 1269 | 31.3% |
| Canada | 10 | 378,446 | 65,603 | 17.3% | 42,106 | 11.1% | 172,004 | 45.5% | 0 | 0.0% | 0 | 0.0% | 98,732 | 26.1% |
| Central African Republic | 6 | 802 | 288 | 35.9% | 138 | 17.2% | 77 | 9.6% | 4 | 0.5% | 150 | 18.7% | 145 | 18.1% |
| Chile | 11 | 32,698 | 11,933 | 36.5% | 8,149 | 24.9% | 4,687 | 14.3% | 3,574 | 10.9% | 1,900 | 5.8% | 2455 | 7.5% |
| China | 10 | 678,837 | 277,333 | 40.9% | 183,578 | 27.0% | 5,869 | 0.9% | 69,486 | 10.2% | 78,362 | 11.5% | 64,100 | 9.4% |
| Colombia | 11 | 8,602 | 2,692 | 31.3% | 4,364 | 50.7% | 319 | 3.7% | 341 | 4.0% | 108 | 1.3% | 778 | 9.0% |
| Congo, Democratic Republic | 10 | 1,530 | 514 | 33.6% | 392 | 25.6% | 70 | 4.6% | 115 | 7.5% | 21 | 1.4% | 418 | 27.3% |
| Costa Rica | 11 | 5,108 | 1,978 | 38.7% | 2,076 | 40.6% | 83 | 1.6% | 8 | 0.2% | 62 | 1.2% | 901 | 17.6% |
| Croatia | 10 | 17,042 | 2,095 | 12.3% | 3,192 | 18.7% | 8,817 | 51.7% | 498 | 2.9% | 184 | 1.1% | 2,256 | 13.2% |
| Cuba | 11 | 5,252 | 1,503 | 28.6% | 2,521 | 48.0% | 161 | 3.1% | 216 | 4.1% | 236 | 4.5% | 614 | 11.7% |
| Cyprus | 4 | 1,037 | 134 | 12.9% | 146 | 14.1% | 593 | 57.2% | 17 | 1.6% | 22 | 2.1% | 125 | 12.1% |
| Czechia | 9 | 4,453 | 1,348 | 30.3% | 1,485 | 33.3% | 381 | 8.6% | 409 | 9.2% | 126 | 2.8% | 704 | 15.8% |
| Denmark | 10 | 54,311 | 7,343 | 13.5% | 6,189 | 11.4% | 22,483 | 41.4% | 4,180 | 7.7% | 1,204 | 2.2% | 12,912 | 23.8% |
| Dominican Republic | 10 | 2,015 | 349 | 17.3% | 1,102 | 54.7% | 14 | 0.7% | 299 | 14.8% | 118 | 5.9% | 133 | 6.6% |
| Ecuador | 11 | 6,187 | 1,697 | 27.4% | 3,495 | 56.5% | 203 | 3.3% | 16 | 0.3% | 32 | 0.5% | 726 | 11.7% |
| Egypt | 10 | 15,680 | 3,588 | 22.9% | 7,647 | 48.8% | 501 | 3.2% | 13 | 0.1% | 16 | 0.1% | 3,915 | 25.0% |
| El Salvador | 10 | 2,300 | 728 | 31.7% | 822 | 35.7% | 81 | 3.5% | 96 | 4.2% | 90 | 3.9% | 483 | 21.0% |
| Estonia | 10 | 11,031 | 465 | 4.2% | 1,108 | 10.0% | 6,663 | 60.4% | 93 | 0.8% | 68 | 0.6% | 2,634 | 23.9% |
| Ethiopia | 7 | 1,273 | 472 | 37.1% | 430 | 33.8% | 2 | 0.2% | 26 | 2.0% | 0 | 0.0% | 343 | 26.9% |
| Fiji | 6 | 679 | 229 | 33.7% | 130 | 19.1% | 30 | 4.4% | 8 | 1.2% | 50 | 7.4% | 232 | 34.2% |
| Finland | 10 | 2,148 | 783 | 36.5% | 832 | 38.7% | 23 | 1.1% | 219 | 10.2% | 69 | 3.2% | 222 | 10.3% |
| France | 10 | 183,788 | 19,201 | 10.4% | 20,324 | 11.1% | 90,357 | 49.2% | 2,896 | 1.6% | 3,313 | 1.8% | 47,697 | 26.0% |
| French Guiana | 10 | 1,625 | 576 | 35.4% | 508 | 31.3% | 6 | 0.4% | 318 | 19.6% | 158 | 9.7% | 59 | 3.6% |
| Georgia | 9 | 2,385 | 458 | 19.2% | 1,156 | 48.5% | 15 | 0.6% | 0 | 0.0% | 128 | 5.4% | 628 | 26.3% |
| Germany | 10 | 14,067 | 5,080 | 36.1% | 4,553 | 32.4% | 9 | 0.1% | 2,786 | 19.8% | 1,639 | 11.7% | 0 | 0.0% |
| Ghana | 11 | 6,212 | 2,221 | 35.8% | 2,287 | 36.8% | 25 | 0.4% | 551 | 8.9% | 851 | 13.7% | 277 | 4.5% |
| Greece | 10 | 12,792 | 2,324 | 18.2% | 8,517 | 66.6% | 209 | 1.6% | 347 | 2.7% | 209 | 1.6% | 1,186 | 9.3% |
| Guadeloupe | 6 | 567 | 318 | 56.1% | 158 | 27.9% | 5 | 0.9% | 62 | 10.9% | 12 | 2.1% | 12 | 2.1% |
| Guatemala | 11 | 2,233 | 621 | 27.8% | 878 | 39.3% | 267 | 12.0% | 28 | 1.3% | 125 | 5.6% | 314 | 14.1% |
| Guinea | 2 | 198 | 113 | 57.1% | 5 | 2.5% | 0 | 0.0% | 0 | 0.0% | 73 | 36.9% | 7 | 3.5% |
| Haiti | 5 | 953 | 371 | 38.9% | 408 | 42.8% | 10 | 1.0% | 96 | 10.1% | 63 | 6.6% | 5 | 0.5% |
| Honduras | 10 | 2,101 | 663 | 31.6% | 844 | 40.2% | 19 | 0.9% | 11 | 0.5% | 71 | 3.4% | 492 | 23.4% |
| Hungary | 10 | 5,919 | 1,874 | 31.7% | 1,635 | 27.6% | 724 | 12.2% | 618 | 10.4% | 426 | 7.2% | 642 | 10.8% |
| Iceland | 10 | 3,323 | 1,570 | 47.2% | 802 | 24.1% | 16 | 0.5% | 0 | 0.0% | 126 | 3.8% | 809 | 24.3% |
| India | 11 | 34,068 | 6,265 | 18.4% | 23,223 | 68.2% | 2 | 0.0% | 248 | 0.7% | 624 | 1.8% | 3,705 | 10.9% |
| Indonesia | 11 | 9,384 | 3,263 | 34.8% | 2,306 | 24.6% | 77 | 0.8% | 0 | 0.0% | 22 | 0.2% | 3,716 | 39.6% |
| Iran | 10 | 19,073 | 4,930 | 25.8% | 7,690 | 40.3% | 2,730 | 14.3% | 18 | 0.1% | 2 | 0.0% | 3,703 | 19.4% |
| Iraq | 8 | 2,813 | 191 | 6.8% | 1,837 | 65.3% | 371 | 13.2% | 0 | 0.0% | 0 | 0.0% | 414 | 14.7% |
| Ireland | 10 | 23,065 | 9,381 | 40.7% | 6,398 | 27.7% | 618 | 2.7% | 2 | 0.0% | 121 | 0.5% | 6,545 | 28.4% |
| Israel | 10 | 13,522 | 2,172 | 16.1% | 4,290 | 31.7% | 3,464 | 25.6% | 28 | 0.2% | 1 | 0.0% | 3,567 | 26.4% |
| Italy | 10 | 30,559 | 10,335 | 33.8% | 10,388 | 34.0% | 1,938 | 6.3% | 1,752 | 5.7% | 925 | 3.0% | 5,217 | 17.1% |
| Ivory Coast | 11 | 3,385 | 1,035 | 30.6% | 832 | 24.6% | 240 | 7.1% | 264 | 7.8% | 511 | 15.1% | 498 | 14.7% |
| Jamaica | 8 | 1,209 | 406 | 33.6% | 521 | 43.1% | 18 | 1.5% | 55 | 4.5% | 78 | 6.5% | 128 | 10.6% |
| Japan | 10 | 82,589 | 37,545 | 45.5% | 24,638 | 29.8% | 69 | 0.1% | 11,113 | 13.5% | 7,509 | 9.1% | 1,715 | 2.1% |
| Jordan | 10 | 2,640 | 619 | 23.4% | 1,554 | 58.9% | 3 | 0.1% | 61 | 2.3% | 102 | 3.9% | 301 | 11.4% |
| Kazakhstan | 10 | 6,079 | 2,568 | 42.2% | 1,938 | 31.9% | 116 | 1.9% | 5 | 0.1% | 2 | 0.0% | 1,450 | 23.9% |
| Kenya | 9 | 3,671 | 1,308 | 35.6% | 1,079 | 29.4% | 201 | 5.5% | 13 | 0.4% | 214 | 5.8% | 856 | 23.3% |
| Kosovo | 4 | 1,320 | 5 | 0.4% | 38 | 2.9% | 784 | 59.4% | 9 | 0.7% | 10 | 0.8% | 474 | 35.9% |
| Kuwait | 3 | 13,104 | 924 | 7.1% | 5,767 | 44.0% | 3,452 | 26.3% | 45 | 0.3% | 549 | 4.2% | 2,360 | 18.0% |
| Kyrgyzstan | 8 | 1,481 | 494 | 33.4% | 333 | 22.5% | 4 | 0.3% | 101 | 6.8% | 185 | 12.5% | 364 | 24.6% |
| Lao | 10 | 4,596 | 1,634 | 35.6% | 1,231 | 26.8% | 4 | 0.1% | 337 | 7.3% | 697 | 15.2% | 693 | 15.1% |
| Latvia | 10 | 19,746 | 1,231 | 6.2% | 1,393 | 7.1% | 11,124 | 56.3% | 530 | 2.7% | 275 | 1.4% | 5,193 | 26.3% |
| Lebanon | 6 | 1,155 | 350 | 30.3% | 287 | 24.8% | 0 | 0.0% | 92 | 8.0% | 209 | 18.1% | 217 | 18.8% |
| Lithuania | 10 | 7,302 | 2,381 | 32.6% | 2,830 | 38.8% | 466 | 6.4% | 689 | 9.4% | 404 | 5.5% | 532 | 7.3% |
| Luxembourg | 10 | 4,518 | 1,076 | 23.8% | 1,453 | 32.2% | 252 | 5.6% | 0 | 0.0% | 0 | 0.0% | 1,737 | 38.4% |
| Madagascar | 10 | 4,700 | 1,344 | 28.6% | 892 | 19.0% | 488 | 10.4% | 239 | 5.1% | 463 | 9.9% | 1,274 | 27.1% |
| Malaysia | 9 | 3,297 | 581 | 17.6% | 1,064 | 32.3% | 452 | 13.7% | 18 | 0.5% | 29 | 0.9% | 1,153 | 35.0% |
| Maldives | 3 | 910 | 329 | 36.2% | 366 | 40.2% | 6 | 0.7% | 47 | 5.2% | 23 | 2.5% | 139 | 15.3% |
| Mali | 9 | 1,182 | 447 | 37.8% | 301 | 25.5% | 10 | 0.8% | 20 | 1.7% | 157 | 13.3% | 247 | 20.9% |
| Malta | 8 | 3,710 | 1,061 | 28.6% | 1,282 | 34.6% | 203 | 5.5% | 1 | 0.0% | 5 | 0.1% | 1,158 | 31.2% |
| Martinique | 6 | 994 | 387 | 38.9% | 350 | 35.2% | 5 | 0.5% | 182 | 18.3% | 68 | 6.8% | 2 | 0.2% |
| Mauritania | 1 | 51 | 30 | 58.8% | 11 | 21.6% | 3 | 5.9% | 0 | 0.0% | 0 | 0.0% | 7 | 13.7% |
| Mauritius | 10 | 1,369 | 383 | 28.0% | 623 | 45.5% | 174 | 12.7% | 0 | 0.0% | 0 | 0.0% | 189 | 13.8% |
| Mexico | 10 | 57,154 | 17,030 | 29.8% | 25,905 | 45.3% | 1,806 | 3.2% | 1,500 | 2.6% | 2,302 | 4.0% | 7,319 | 12.8% |
| Moldova | 9 | 2,493 | 547 | 21.9% | 1,234 | 49.5% | 38 | 1.5% | 87 | 3.5% | 30 | 1.2% | 557 | 22.3% |
| Mongolia | 10 | 5,692 | 2,729 | 47.9% | 1,424 | 25.0% | 142 | 2.5% | 318 | 5.6% | 388 | 6.8% | 676 | 11.9% |
| Montenegro | 5 | 1,588 | 27 | 1.7% | 312 | 19.6% | 853 | 53.7% | 129 | 8.1% | 21 | 1.3% | 246 | 15.5% |
| Morocco | 9 | 2,898 | 1,015 | 35.0% | 1,027 | 35.4% | 11 | 0.4% | 161 | 5.6% | 59 | 2.0% | 625 | 21.6% |
| Mozambique | 3 | 235 | 79 | 33.6% | 53 | 22.6% | 17 | 7.2% | 41 | 17.4% | 43 | 18.3% | 2 | 0.9% |
| Myanmar | 4 | 1,740 | 310 | 17.8% | 979 | 56.3% | 0 | 0.0% | 27 | 1.6% | 1 | 0.1% | 423 | 24.3% |
| Nepal | 9 | 12,122 | 3,433 | 28.3% | 5,099 | 42.1% | 20 | 0.2% | 472 | 3.9% | 112 | 0.9% | 2,986 | 24.6% |
| Netherlands | 10 | 26,509 | 6,982 | 26.3% | 4,325 | 16.3% | 9,895 | 37.3% | 2,459 | 9.3% | 1,145 | 4.3% | 1,703 | 6.4% |
| New Caledonia | 10 | 2,162 | 760 | 35.2% | 679 | 31.4% | 23 | 1.1% | 154 | 7.1% | 173 | 8.0% | 373 | 17.3% |
| New Zealand | 10 | 17,235 | 4,468 | 25.9% | 2,602 | 15.1% | 4,463 | 25.9% | 642 | 3.7% | 582 | 3.4% | 4,478 | 26.0% |
| Nicaragua | 10 | 7,975 | 2,765 | 34.7% | 2,610 | 32.7% | 153 | 1.9% | 18 | 0.2% | 0 | 0.0% | 2,429 | 30.5% |
| Niger | 7 | 710 | 215 | 30.3% | 242 | 34.1% | 22 | 3.1% | 13 | 1.8% | 88 | 12.4% | 130 | 18.3% |
| Nigeria | 9 | 1,241 | 278 | 22.4% | 314 | 25.3% | 214 | 17.2% | 2 | 0.2% | 25 | 2.0% | 408 | 32.9% |
| North Korea | 4 | 973 | 191 | 19.6% | 691 | 71.0% | 0 | 0.0% | 0 | 0.0% | 0 | 0.0% | 91 | 9.4% |
| North Macedonia | 3 | 944 | 122 | 12.9% | 513 | 54.3% | 78 | 8.3% | 116 | 12.3% | 82 | 8.7% | 33 | 3.5% |
| Norway | 10 | 152,992 | 12,338 | 8.1% | 18,035 | 11.8% | 70,615 | 46.2% | 4,648 | 3.0% | 2,308 | 1.5% | 45,048 | 29.4% |
| Oman | 11 | 11,316 | 2,624 | 23.2% | 5,126 | 45.3% | 646 | 5.7% | 79 | 0.7% | 327 | 2.9% | 2,514 | 22.2% |
| Pakistan | 9 | 3,718 | 854 | 23.0% | 1,900 | 51.1% | 466 | 12.5% | 73 | 2.0% | 66 | 1.8% | 359 | 9.7% |
| Panama | 10 | 2,544 | 749 | 29.4% | 1,326 | 52.1% | 22 | 0.9% | 148 | 5.8% | 140 | 5.5% | 159 | 6.3% |
| Papua New Guinea | 1 | 144 | 7 | 4.9% | 106 | 73.6% | 4 | 2.8% | 15 | 10.4% | 8 | 5.6% | 4 | 2.8% |
| Paraguay | 11 | 9,206 | 3,888 | 42.2% | 2,999 | 32.6% | 188 | 2.0% | 76 | 0.8% | 56 | 0.6% | 1,999 | 21.7% |
| Peru | 11 | 7,688 | 1,529 | 19.9% | 3,801 | 49.4% | 872 | 11.3% | 585 | 7.6% | 175 | 2.3% | 726 | 9.4% |
| Philippines | 10 | 6,018 | 1,446 | 24.0% | 1,537 | 25.5% | 384 | 6.4% | 837 | 13.9% | 538 | 8.9% | 1,273 | 21.2% |
| Poland | 9 | 14,404 | 446 | 3.1% | 5,379 | 37.3% | 5,143 | 35.7% | 0 | 0.0% | 6 | 0.0% | 3,430 | 23.8% |
| Portugal | 10 | 22,620 | 4,814 | 21.3% | 5,341 | 23.6% | 6,806 | 30.1% | 872 | 3.9% | 291 | 1.3% | 4,496 | 19.9% |
| Qatar | 10 | 44,488 | 3,461 | 7.8% | 14,911 | 33.5% | 14,654 | 32.9% | 1 | 0.0% | 0 | 0.0% | 11,461 | 25.8% |
| Romania | 10 | 11,799 | 2,568 | 21.8% | 4,476 | 37.9% | 915 | 7.8% | 18 | 0.2% | 6 | 0.1% | 3,814 | 32.3% |
| Russia | 10 | 183,366 | 53,068 | 28.9% | 80,962 | 44.2% | 7,710 | 4.2% | 14 | 0.0% | 14 | 0.0% | 41,598 | 22.7% |
| Rwanda | 7 | 722 | 306 | 42.4% | 304 | 42.1% | 0 | 0.0% | 15 | 2.1% | 0 | 0.0% | 97 | 13.4% |
| Saudi Arabia | 4 | 4,947 | 0 | 0.0% | 1,955 | 39.5% | 1,679 | 33.9% | 0 | 0.0% | 0 | 0.0% | 1,313 | 26.5% |
| Senegal | 11 | 7,283 | 2,984 | 41.0% | 1,716 | 23.6% | 13 | 0.2% | 338 | 4.6% | 772 | 10.6% | 1,458 | 20.0% |
| Serbia | 10 | 6,275 | 1,681 | 26.8% | 3,068 | 48.9% | 160 | 2.5% | 377 | 6.0% | 173 | 2.8% | 816 | 13.0% |
| Sierra Leone | 2 | 138 | 75 | 54.3% | 29 | 21.0% | 0 | 0.0% | 0 | 0.0% | 0 | 0.0% | 34 | 24.6% |
| Singapore | 11 | 12,953 | 4,481 | 34.6% | 4,644 | 35.9% | 134 | 1.0% | 1,322 | 10.2% | 1,073 | 8.3% | 1,299 | 10.0% |
| Slovakia | 10 | 3,398 | 688 | 20.2% | 1,061 | 31.2% | 420 | 12.4% | 478 | 14.1% | 162 | 4.8% | 589 | 17.3% |
| Slovenia | 10 | 25,722 | 2,586 | 10.1% | 2,476 | 9.6% | 13,390 | 52.1% | 1,131 | 4.4% | 707 | 2.7% | 5,432 | 21.1% |
| South Africa | 11 | 11,903 | 4,609 | 38.7% | 3,824 | 32.1% | 77 | 0.6% | 491 | 4.1% | 886 | 7.4% | 2,016 | 16.9% |
| South Korea | 10 | 18,417 | 7,293 | 39.6% | 4,768 | 25.9% | 1 | 0.0% | 0 | 0.0% | 0 | 0.0% | 6,355 | 34.5% |
| Spain | 10 | 103,902 | 24,444 | 23.5% | 21,574 | 20.8% | 31,150 | 30.0% | 333 | 0.3% | 113 | 0.1% | 26,282 | 25.3% |
| Sri Lanka | 11 | 9,839 | 1,354 | 13.8% | 2,802 | 28.5% | 2,934 | 29.8% | 0 | 0.0% | 0 | 0.0% | 2,608 | 26.5% |
| Suriname | 3 | 291 | 47 | 16.2% | 216 | 74.2% | 2 | 0.7% | 1 | 0.3% | 8 | 2.7% | 17 | 5.8% |
| Sweden | 10 | 98,447 | 5,478 | 5.6% | 9,287 | 9.4% | 54,359 | 55.2% | 775 | 0.8% | 680 | 0.7% | 27,868 | 28.3% |
| Switzerland | 10 | 65,414 | 2,110 | 3.2% | 3,018 | 4.6% | 42,248 | 64.6% | 977 | 1.5% | 1,058 | 1.6% | 16,003 | 24.5% |
| Tajikistan | 1 | 62 | 0 | 0.0% | 0 | 0.0% | 9 | 14.5% | 0 | 0.0% | 0 | 0.0% | 53 | 85.5% |
| Tanzania | 11 | 2,302 | 927 | 40.3% | 544 | 23.6% | 133 | 5.8% | 7 | 0.3% | 0 | 0.0% | 691 | 30.0% |
| Thailand | 11 | 10,509 | 3,215 | 30.6% | 3,648 | 34.7% | 12 | 0.1% | 807 | 7.7% | 1,237 | 11.8% | 1,590 | 15.1% |
| Timor-Leste | 2 | 372 | 36 | 9.7% | 145 | 39.0% | 0 | 0.0% | 13 | 3.5% | 178 | 47.8% | 0 | 0.0% |
| Togo | 10 | 1,752 | 678 | 38.7% | 343 | 19.6% | 23 | 1.3% | 0 | 0.0% | 0 | 0.0% | 708 | 40.4% |
| Trinidad and Tobago | 1 | 270 | 29 | 10.7% | 240 | 88.9% | 1 | 0.4% | 0 | 0.0% | 0 | 0.0% | 0 | 0.0% |
| Tunisia | 10 | 2,647 | 690 | 26.1% | 1,416 | 53.5% | 2 | 0.1% | 77 | 2.9% | 0 | 0.0% | 462 | 17.5% |
| Turkey | 10 | 25,219 | 8,083 | 32.1% | 10,575 | 41.9% | 63 | 0.2% | 4 | 0.0% | 178 | 0.7% | 6,316 | 25.0% |
| Uganda | 9 | 3,100 | 1,215 | 39.2% | 636 | 20.5% | 362 | 11.7% | 0 | 0.0% | 0 | 0.0% | 887 | 28.6% |
| UK | 10 | 176,557 | 26,637 | 15.1% | 19,797 | 11.2% | 86,353 | 48.9% | 1,516 | 0.9% | 517 | 0.3% | 41,737 | 23.6% |
| Ukraine | 10 | 16,543 | 2,950 | 17.8% | 5,193 | 31.4% | 3,556 | 21.5% | 75 | 0.5% | 101 | 0.6% | 4,668 | 28.2% |
| United Arab Emirates | 1 | 183 | 27 | 14.8% | 84 | 45.9% | 0 | 0.0% | 0 | 0.0% | 13 | 7.1% | 59 | 32.2% |
| Uruguay | 9 | 1,290 | 558 | 43.3% | 558 | 43.3% | 7 | 0.5% | 66 | 5.1% | 64 | 5.0% | 37 | 2.9% |
| USA | 10 | 1,437,794 | 210,430 | 14.6% | 113,432 | 7.9% | 715,151 | 49.7% | 21,747 | 1.5% | 21,645 | 1.5% | 355,096 | 24.7% |
| Uzbekistan | 4 | 387 | 104 | 26.9% | 127 | 32.8% | 2 | 0.5% | 0 | 0.0% | 0 | 0.0% | 154 | 39.8% |
| Venezuela | 6 | 3,698 | 226 | 6.1% | 2,539 | 68.7% | 805 | 21.8% | 57 | 1.5% | 24 | 0.6% | 47 | 1.3% |
| Viet Nam | 11 | 7,230 | 2,397 | 33.2% | 2,307 | 31.9% | 13 | 0.2% | 19 | 0.3% | 61 | 0.8% | 2,423 | 33.5% |
| West Bank and Gaza Strip | 3 | 1,888 | 611 | 32.4% | 1,171 | 62.0% | 7 | 0.4% | 0 | 0.0% | 0 | 0.0% | 99 | 5.2% |
| Yemen | 2 | 315 | 16 | 5.1% | 265 | 84.1% | 13 | 4.1% | 0 | 0.0% | 0 | 0.0% | 21 | 6.7% |
| Zambia | 10 | 1,771 | 515 | 29.1% | 257 | 14.5% | 121 | 6.8% | 0 | 0.0% | 35 | 2.0% | 797 | 45.0% |
| **Total** | **1244** | **4,659,001** | **1,010,978** | **21.7%** | **898,269** | **19.3%** | **1,467,965** | **31.5%** | **152,158** | **3.3%** | **147,118** | **3.2%** | **980,526** | **21.0%** |

**Table S4**. Typical timing and amplitude of the primary and secondary peak, and median duration (in weeks), of influenza epidemics by country (sorted according to the latitude of the country centroid). WHO FluNet database 2010-2019. Only countries with ≥ 5 seasons with ≥ 50 reported influenza cases were included in the analysis. See text for details.

| **Country** | **Latitude** | **N season (≥50 cases)** | **Month primary peak** | **Amplitude primary peak** | **Month secondary peak** | **Amplitude secondary peak** | **Median duration (in weeks)** |
| --- | --- | --- | --- | --- | --- | --- | --- |
| Norway | 69 | 10 | Feb, 2nd half | 102.0% | Jul, 1st half | 6.0% | 11 |
| Iceland | 65 | 10 | Mar, 1st half | 102.0% | Oct, 2nd half | 9.0% | 10 |
| Finland | 65 | 10 | Feb, 2nd half | 106.0% | Oct, 2nd half | 12.0% | 13 |
| Sweden | 63 | 10 | Feb, 2nd half | 102.0% | Jul, 1st half | 6.0% | 11 |
| Russia | 62 | 10 | Mar, 1st half | 105.0% | Oct, 2nd half | 11.0% | 9 |
| Canada | 61 | 10 | Feb, 1st half | 103.0% | Sep, 1st half | 8.0% | 13 |
| Estonia | 59 | 10 | Feb, 2nd half | 106.0% | Jun, 2nd half | 12.0% | 9 |
| Latvia | 57 | 10 | Mar, 1st half | 105.0% | Oct, 2nd half | 8.0% | 9 |
| Denmark | 56 | 10 | Mar, 1st half | 108.0% | Oct, 2nd half | 15.0% | 9 |
| Lithuania | 55 | 10 | Feb, 2nd half | 111.0% | Oct, 1st half | 20.0% | 7 |
| UK | 54 | 10 | Feb, 1st half | 102.0% | Sep, 2nd half | 7.0% | 12 |
| Belarus | 54 | 10 | Feb, 2nd half | 111.0% | Jul, 1st half | 19.0% | 7 |
| Ireland | 53 | 10 | Feb, 1st half | 104.0% | Sep, 2nd half | 9.0% | 10 |
| Poland | 52 | 9 | Feb, 2nd half | 109.0% | Oct, 2nd half | 15.0% | 7 |
| Netherlands | 52 | 10 | Feb, 2nd half | 104.0% | Oct, 1st half | 9.0% | 10 |
| Germany | 51 | 10 | Feb, 2nd half | 106.0% | Oct, 2nd half | 13.0% | 9 |
| Belgium | 51 | 10 | Feb, 2nd half | 107.0% | Jul, 1st half | 14.0% | 8 |
| Luxembourg | 50 | 10 | Feb, 2nd half | 108.0% | Jul, 1st half | 15.0% | 8 |
| Czechia | 50 | 9 | Feb, 2nd half | 106.0% | Oct, 1st half | 14.0% | 8 |
| Ukraine | 49 | 10 | Mar, 1st half | 105.0% | Jul, 2nd half | 7.0% | 9 |
| Slovakia | 49 | 10 | Mar, 1st half | 111.0% | Oct, 2nd half | 19.0% | 8 |
| Kazakhstan | 48 | 10 | Feb, 2nd half | 108.0% | Oct, 1st half | 15.0% | 8 |
| Austria | 48 | 10 | Feb, 2nd half | 111.0% | Oct, 1st half | 18.0% | 8 |
| Hungary | 47 | 10 | Feb, 2nd half | 111.0% | Oct, 2nd half | 20.0% | 7 |
| Moldova | 47 | 9 | Feb, 2nd half | 108.0% | Jul, 1st half | 15.0% | 7 |
| Mongolia | 47 | 10 | Feb, 1st half | 105.0% | Sep, 2nd half | 13.0% | 8 |
| Switzerland | 47 | 10 | Feb, 2nd half | 108.0% | Oct, 1st half | 14.0% | 8 |
| Slovenia | 46 | 10 | Feb, 2nd half | 108.0% | Oct, 1st half | 15.0% | 8 |
| Romania | 46 | 10 | Mar, 1st half | 108.0% | Oct, 2nd half | 16.0% | 8 |
| Croatia | 45 | 10 | Feb, 2nd half | 105.0% | Sep, 2nd half | 8.0% | 9 |
| Serbia | 44 | 10 | Feb, 2nd half | 109.0% | Oct, 1st half | 17.0% | 7 |
| Italy | 43 | 10 | Feb, 2nd half | 110.0% | Oct, 1st half | 17.0% | 7 |
| Montenegro | 43 | 5 | Feb, 2nd half | 114.0% | Oct, 2nd half | 21.0% | 7 |
| Bulgaria | 43 | 10 | Feb, 1st half | 113.0% | Oct, 1st half | 23.0% | 6 |
| France | 42 | 10 | Feb, 2nd half | 107.0% | Oct, 1st half | 13.0% | 8 |
| Georgia | 42 | 9 | Feb, 2nd half | 109.0% | Jun, 2nd half | 17.0% | 8 |
| Kyrgyzstan | 42 | 8 | Jan, 2nd half | 106.0% | Aug, 2nd half | 12.0% | 6 |
| Albania | 41 | 10 | Feb, 2nd half | 112.0% | Oct, 1st half | 20.0% | 6 |
| Armenia | 40 | 6 | Feb, 1st half | 106.0% | Oct, 1st half | 15.0% | 9 |
| Azerbaijan | 40 | 5 | Mar, 1st half | 109.0% | Jul, 2nd half | 15.0% | 7 |
| Spain | 40 | 10 | Feb, 1st half | 108.0% | Sep, 2nd half | 14.0% | 7 |
| Portugal | 40 | 10 | Feb, 1st half | 106.0% | Sep, 2nd half | 12.0% | 8 |
| Greece | 39 | 10 | Feb, 2nd half | 116.0% | Jun, 2nd half | 27.0% | 7 |
| Turkey | 39 | 10 | Feb, 1st half | 112.0% | May, 2nd half | 21.0% | 8 |
| USA | 38 | 10 | Feb, 1st half | 99.0% | Sep, 2nd half | 4.0% | 13 |
| Japan | 38 | 10 | Feb, 1st half | 104.0% | May, 2nd half | 14.0% | 13 |
| China | 37 | 10 | Feb, 1st half | 93.0% | Aug, 2nd half | 19.0% | 26 |
| South Korea | 36 | 10 | Feb, 1st half | 107.0% | Sep, 1st half | 12.0% | 11 |
| Malta | 36 | 8 | Feb, 1st half | 109.0% | Sep, 2nd half | 20.0% | 9 |
| Tunisia | 34 | 10 | Mar, 1st half | 104.0% | Jul, 2nd half | 8.0% | 8 |
| Lebanon | 34 | 6 | Feb, 2nd half | 102.0% | Aug, 1st half | 7.0% | 10 |
| Iraq | 33 | 8 | Feb, 1st half | 113.0% | Sep, 2nd half | 23.0% | 7 |
| Iran | 32 | 10 | Jan, 1st half | 104.0% | Aug, 2nd half | 12.0% | 9 |
| Israel | 32 | 10 | Feb, 1st half | 105.0% | Sep, 2nd half | 11.0% | 8 |
| Jordan | 31 | 10 | Jan, 1st half | 114.0% | May, 1st half | 60.0% | 8 |
| Pakistan | 30 | 9 | Feb, 1st half | 107.0% | Oct, 1st half | 20.0% | 8 |
| Morocco | 30 | 9 | Feb, 1st half | 107.0% | Jun, 2nd half | 12.0% | 10 |
| Nepal | 28 | 9 | Aug, 2nd half | 107.0% | Mar, 1st half | 78.0% | 14 |
| Algeria | 28 | 9 | Feb, 1st half | 105.0% | Jun, 2nd half | 11.0% | 9 |
| Bhutan | 27 | 9 | Aug, 2nd half | 80.0% | Apr, 1st half | 79.0% | 25 |
| Egypt | 27 | 10 | Jan, 1st half | 100.0% | May, 1st half | 26.0% | 21 |
| Bahrain | 26 | 8 | Nov, 2nd half | 108.0% | Mar, 1st half | 45.0% | 23 |
| Qatar | 25 | 10 | Dec, 1st half | 89.0% | Jul, 1st half | 3.0% | 24 |
| Mexico | 24 | 10 | Feb, 2nd half | 99.0% | Oct, 2nd half | 15.0% | 14 |
| Bangladesh | 24 | 10 | Jul, 1st half | 99.0% | not defined | 0.0% | 42 |
| India | 23 | 10 | Mar, 1st half | 97.0% | Sep, 1st half | 52.0% | 26 |
| Cuba | 22 | 10 | Aug, 1st half | 79.0% | not defined | 0.0% | 25 |
| Oman | 21 | 10 | Nov, 2nd half | 91.0% | Mar, 2nd half | 49.0% | 37 |
| Dominican Republic | 19 | 9 | Apr, 2nd half | 88.0% | Nov, 2nd half | 34.0% | 23 |
| Jamaica | 18 | 7 | Mar, 1st half | 98.0% | Oct, 2nd half | 49.0% | 25 |
| Laos | 18 | 9 | Oct, 2nd half | 82.0% | Feb, 1st half | 33.0% | 32 |
| Niger | 17 | 6 | Feb, 2nd half | 101.0% | Oct, 2nd half | 46.0% | 34 |
| Mali | 17 | 8 | Feb, 2nd half | 105.0% | Oct, 2nd half | 93.0% | 32 |
| Guadeloupe | 16 | 6 | Feb, 2nd half | 109.0% | Jul, 1st half | 17.0% | 9 |
| Viet Nam | 16 | 10 | Jul, 2nd half | 63.0% | Nov, 1st half | 54.0% | 30 |
| Guatemala | 16 | 10 | Mar, 2nd half | 100.0% | Jul, 2nd half | 36.0% | 23 |
| Thailand | 15 | 10 | Sep, 2nd half | 84.0% | Mar, 1st half | 50.0% | 34 |
| Honduras | 15 | 9 | Aug, 1st half | 77.0% | Oct, 2nd half | 73.0% | 21 |
| Martinique | 14 | 6 | Mar, 1st half | 105.0% | Nov, 1st half | 13.0% | 24 |
| Senegal | 14 | 10 | Oct, 2nd half | 108.0% | Mar, 1st half | 38.0% | 25 |
| El Salvador | 14 | 10 | Jun, 2nd half | 96.0% | Oct, 2nd half | 58.0% | 16 |
| Nicaragua | 13 | 10 | Oct, 2nd half | 100.0% | Mar, 1st half | 12.0% | 11 |
| Cambodia | 13 | 10 | Nov, 1st half | 97.0% | Jul, 1st half | 33.0% | 21 |
| Burkina Faso | 12 | 6 | Feb, 1st half | 103.0% | Oct, 2nd half | 36.0% | 40 |
| Philippines | 12 | 10 | Sep, 1st half | 93.0% | Mar, 1st half | 26.0% | 27 |
| Costa Rica | 10 | 10 | Dec, 1st half | 88.0% | Jul, 2nd half | 83.0% | 27 |
| Nigeria | 10 | 9 | Oct, 2nd half | 74.0% | Feb, 2nd half | 70.0% | 28 |
| Ethiopia | 9 | 7 | Mar, 1st half | 76.0% | Nov, 2nd half | 56.0% | 35 |
| Togo | 9 | 9 | Nov, 1st half | 96.0% | Jul, 1st half | 34.0% | 27 |
| Panama | 9 | 9 | Jun, 2nd half | 112.0% | Nov, 1st half | 34.0% | 11 |
| Ivory Coast | 8 | 10 | Nov, 1st half | 77.0% | Jul, 1st half | 59.0% | 28 |
| Venezuela | 8 | 6 | Apr, 1st half | 115.0% | Nov, 2nd half | 52.0% | 22 |
| Ghana | 8 | 10 | Nov, 2nd half | 90.0% | Apr, 1st half | 72.0% | 23 |
| Sri Lanka | 8 | 10 | Jun, 2nd half | 92.0% | Dec, 1st half | 84.0% | 28 |
| Central African Republic | 7 | 6 | Sep, 1st half | 99.0% | Jan, 1st half | 11.0% | 10 |
| Cameroon | 6 | 10 | Nov, 2nd half | 90.0% | Apr, 1st half | 19.0% | 19 |
| French Guiana | 4 | 9 | Mar, 1st half | 90.0% | May, 2nd half | 68.0% | 19 |
| Colombia | 4 | 10 | Jun, 2nd half | 70.0% | Feb, 1st half | 17.0% | 33 |
| Malaysia | 4 | 8 | Apr, 1st half | 48.0% | not defined | 0.0% | 36 |
| Singapore | 1 | 10 | Jun, 2nd half | 68.0% | Jan, 2nd half | 40.0% | 33 |
| Uganda | 1 | 9 | Aug, 2nd half | 100.0% | May, 1st half | 52.0% | 19 |
| Kenya | 1 | 8 | Mar, 2nd half | 72.0% | Nov, 2nd half | 57.0% | 34 |
| Ecuador | -1 | 10 | Jan, 2nd half | 105.0% | Sep, 1st half | 43.0% | 33 |
| Rwanda | -2 | 7 | Apr, 1st half | 105.0% | Nov, 1st half | 19.0% | 11 |
| Indonesia | -2 | 10 | Feb, 2nd half | 85.0% | Oct, 2nd half | 27.0% | 36 |
| Congo, Democratic Republic | -3 | 10 | Jan, 2nd half | 95.0% | May, 2nd half | 86.0% | 27 |
| Tanzania | -6 | 10 | Feb, 1st half | 87.0% | May, 1st half | 78.0% | 34 |
| Peru | -9 | 10 | Aug, 1st half | 84.0% | Dec, 1st half | 12.0% | 21 |
| Brazil | -11 | 10 | May, 2nd half | 89.0% | Oct, 2nd half | 8.0% | 25 |
| Zambia | -14 | 10 | Aug, 2nd half | 72.0% | Apr, 1st half | 61.0% | 26 |
| Bolivia | -17 | 10 | Jun, 1st half | 98.0% | Oct, 1st half | 50.0% | 11 |
| Fiji | -17 | 6 | May, 2nd half | 112.0% | Oct, 1st half | 73.0% | 12 |
| Madagascar | -19 | 10 | Feb, 2nd half | 64.0% | Jun, 2nd half | 39.0% | 30 |
| Mauritius | -20 | 9 | Jul, 2nd half | 103.0% | Mar, 1st half | 90.0% | 25 |
| New Caledonia | -21 | 10 | Aug, 2nd half | 92.0% | Apr, 1st half | 76.0% | 17 |
| Paraguay | -23 | 10 | Jul, 1st half | 104.0% | Nov, 2nd half | 21.0% | 13 |
| Australia | -26 | 10 | Sep, 1st half | 99.0% | Feb, 1st half | 16.0% | 16 |
| South Africa | -29 | 10 | Jul, 1st half | 107.0% | Feb, 1st half | 16.0% | 13 |
| Uruguay | -33 | 9 | Jul, 2nd half | 102.0% | Nov, 2nd half | 17.0% | 7 |
| Argentina | -35 | 10 | Jul, 1st half | 103.0% | Feb, 2nd half | 12.0% | 11 |
| Chile | -38 | 10 | Jul, 2nd half | 100.0% | Feb, 2nd half | 11.0% | 14 |
| New Zealand | -42 | 10 | Aug, 2nd half | 106.0% | Jan, 2nd half | 9.0% | 10 |
